# Association of Dietary Inflammatory Index and Dietary Oxidative Balance Score with All-Cause and Disease-Specific Mortality: Findings of 2003–2014 National Health and Nutrition Examination Survey

**DOI:** 10.3390/nu15143148

**Published:** 2023-07-14

**Authors:** Xuanyang Wang, Jinxia Hu, Lin Liu, Yuntao Zhang, Keke Dang, Licheng Cheng, Jia Zhang, Xiaoqing Xu, Ying Li

**Affiliations:** Department of Nutrition and Food Hygiene, The National Key Discipline, School of Public Health, Harbin Medical University, Harbin 150086, China

**Keywords:** inflammation, oxidative stress, dietary inflammatory index, dietary oxidative balance score, mortality, national health and nutrition examination survey

## Abstract

To clarify the effects of dietary inflammatory and pro-oxidative potential, we investigated the impact of the Dietary Inflammation Index (DII) and the Dietary Oxidative Balance Score (DOBS) on all-cause and disease-specific mortality. For DII and DOBS, 17,550 and 24,527 participants were included. Twenty-six and seventeen dietary factors were selected for scoring. Cox proportional hazards regression models were used. DII and DOBS were significantly associated with all-cause, CVD, and cancer mortality in this nationally representative sample of American adults. Compared with the lowest DII, the multivariable-adjusted hazard ratios (95% CI) of all-cause, CVD, and cancer mortality for the highest were 1.49 (1.23–1.80), 1.58 (1.08–2.33), and 1.56 (1.07–2.25). The highest quartile of DOBS was associated with the risk of all-cause death (HR 0.71, 95% CI 0.59–0.86). Pro-inflammatory and pro-oxidative diets were associated with increased risk for all-cause (HR 1.59, 95% CI 1.28–1.97), and CVD (HR 2.29, 95% CI 1.33–3.94) death compared to anti-inflammatory and antioxidant diets. Similar results were observed among the stratification analyses. Inflammation-reducing and oxidative-balancing diets are linked to lower all-cause and CVD mortality. Diets impact health by regulating inflammation and oxidative stress.

## 1. Introduction

Inflammation and oxidative stress are common physiological responses to external stimuli and certain diseases [1]. However, long-term high levels of inflammation and oxidative stress in the body are also considered to be the main causes of the occurrence and development of diseases [2,3]. For example, there is growing evidence that markers of inflammation and oxidative stress, such as C-reactive protein (CRP), are associated with the increased risk of diseases, including diabetes [4], cardiovascular disease (CVD) [5], cancer [6], and dementia. In general, high levels of inflammation and oxidative stress are sufficient to affect the health status of the body and lead to an increased risk of all-cause and disease-specific mortality [7].

In addition to age, gender, genetic factors, environmental factors, the immune system, and lifestyle, diet, which is an important way to obtain nutrients, is also another key factor affecting inflammation and oxidative stress [8]. Epidemiological evidence suggests that dietary patterns play an important role in maintaining homeostasis in inflammation and oxidative stress [9]. Specifically, dietary patterns such as the Mediterranean diet [10] have been reported to reduce the risk of diabetes [11], CVD [12], and certain cancers [13] by reducing inflammation and oxidative stress. And the effects of dietary components on inflammation and oxidative stress can occur through direct or indirect means. Dietary components that have direct effects include nutrients with anti-inflammatory and antioxidant activity, such as beta-carotene [14] and zinc [15], as well as proteins, fats, and cholesterol, that can cause inflammation or oxidation. Indirect dietary components refer to nutrients that can affect the composition and metabolites of gut microbes, such as dietary fiber [16] and probiotics [17]. In addition, bad eating habits such as consuming a high-fat diet, high-sugar diet, or low-fiber diet may also contribute to heightened inflammation and oxidative stress levels [18,19]. Dietary adjustment is one of the most important measures that can be taken to prevent and cure diseases [20]. Its application in diseases has been proven to bring many health benefits [21]. Epidemiological studies suggest that appropriate dietary adjustments have a beneficial impact on preventing and treating inflammation- and oxidative stress-related diseases [22], for example, reducing one’s intake of trans fats [23] and high-glycemic foods [24], as well as increasing one’s intake of monounsaturated and polyunsaturated fatty acids [25], and micronutrients such as zinc [26], copper [27], manganese [28], and vitamins A, C, and E [29].

DII and DOBS are indicators calculated based on the inflammatory and oxidative effects of nutrients in dietary intake, respectively, to evaluate the effects of diet on inflammation and oxidative stress [28,30]. In general, a lower-DII or a higher-DOBS diet includes plenty of fruits, vegetables, nuts, fish, and dietary fiber, which help reduce levels of inflammation or oxidative stress. A higher-DII or a lower-DOBS diet includes a large amount of high-sugar, high-salt, high-fat, high-cholesterol, high-trans fatty acids, and processed foods, leading to increased inflammation or oxidative stress. To date, an increasing number of studies have reported a correlation of high DII values or low DOBS values with poorer health outcomes and higher risk of chronic diseases, including diabetes [31,32], osteoarthritis [33,34], CVD [35], cancer [36,37], and neurodegenerative diseases [38]. So, it seems reasonable to hypothesize that DII and DOBS may work together to affect mortality [39,40]. Although certain studies have evaluated the association of DII and DOBS with mortality, the relationship of dietary inflammation and pro-oxidative potential with the risk of death is unclear.

To examine the above hypothesis, we explored the association of DII and DOBS with all-cause, CVD, and cancer mortality in the U.S. according to the National Health and Nutrition Examination Survey (NHANES) (2003–2014).

## 2. Materials and Methods

### 2.1. Study Population

**Figure 1:**
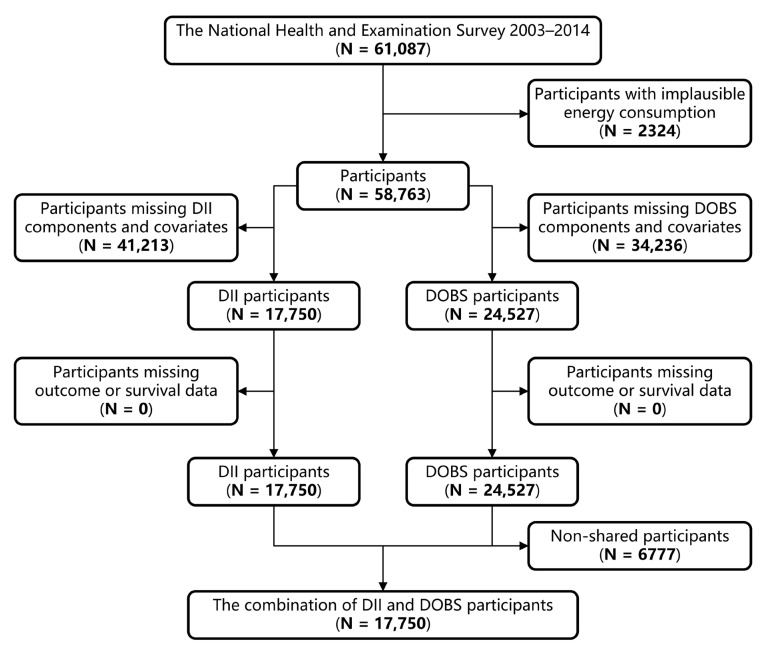
Flowchart of the selection strategy.

NHANES is a stratified, multistage, nationwide study conducted by the National Center for Health Statistics (NCHS) to gain an accurate picture of the health and nutritional status of Americans [41]. Detailed information about NHANES has been previously described [42,43]. This study was performed on the data of adults (age ≥ 18 years) who participated in NHANES (2003–2014). Individuals who met any of the following conditions were excluded: (1) implausible energy consumption (<800 or >4200 kcal/day for men and <500 or >3500 kcal/day for women) [44], (2) missing data for DII/DOBS components or covariates, or (3) missing outcome states of interest. After excluding the above participants, a total of 17,550 and 24,527 individuals were included in this prospective study for DII and DOBS exposure, respectively (Figure 1). Before the survey, all protocols received approval from the Institutional Review Board of the National Center for Health Statistics, and informed consent was obtained from all participants.

### 2.2. Dietary Information

Daily dietary intake was assessed by conducting 24 h dietary recall interviews over two consecutive days. Each dietary nutrient and total dietary energy were calculated with reference to the relevant guidelines of the U.S. Department of Agriculture’s Food and Nutrient Database for Dietary Studies (FNDDS) [45]. Information on dietary supplement usage was assessed via a dedicated questionnaire. Furthermore, various dietary components derived from dietary nutrient supplementation were not included in the nutrient estimates.

### 2.3. Assessment of DII

DII has been described in detail in [46]. This score, derived from the literature review together with population data, includes 45 dietary components associated with dietary inflammatory potential and their representative dietary intake ranges. Specifics regarding the constituents of the diet involved in DII are given in Supplementary Table S1. Twenty-six of the forty-five dietary components were selected for DII calculation. The predictive power of DII has been demonstrated to be stable when using these food parameters [47]. The DII of a certain dietary component was calculated as follows: (daily intake—global average daily intake)/the standard deviation of the global average daily intake * the overall inflammatory effect score of this dietary component. The sum of the DII of each dietary component was the participant’s DII.

### 2.4. Assessment of DOBS

DOBS was determined by combining the scores of pre-determined pro- and anti-oxidant factors, and took into account both pro- and anti-oxidant factors in the diet [30,48]. Based on prior information on the relationships between certain nutrients and DOBS, a total of 17 nutrients were included in the calculation of DOBS, including 3 pro-oxidants and 14 antioxidants. Most of these food components have been proven several times in existing studies to be useful in the calculation of DOBS [49,50]. After classifying the continuous dietary variable into tertiles, antioxidants were scored from 1 to 3, and pro-oxidants were scored inversely. Regarding alcohol intake, non-drinkers (0 g/d), non-heavy drinkers (0 to 30 g/d for male and 0 to 15 g/d for female), and heavy drinkers (>30 g/d for male and >15 g/d for female) were given scores of 3, 2, and 1, respectively [50]. Meanwhile, vitamin C, beta-carotene, and vitamin B12 were ln-transformed to better approximate normal distributions. A detailed setup scheme for the DOBS components can be found within Supplementary Table S2. The sum of each dietary ingredient score selected was the participant’s DOBS.

### 2.5. Multivariate Linear Regression Models

In the validation sub-study, the relationships of DII/DOBS with CRP and red cell distribution width (RDW) were assessed after adjusting for the same covariates as in the CPH regression models. RDW is a measure of the variation of circulating erythrocyte volume and is primarily used as an indicator to help identify and classify different types of anemia. Similar to CRP, elevated RDW is a potential parameter characterizing ongoing inflammation and has been shown to be correlated with the level of inflammatory markers in patients with rheumatoid arthritis, CVD, and Crohn’s disease [51,52].

### 2.6. Main Outcomes

The outcome variables for this study included mortality in all participants that occurred after their participation in the survey and before 31 December 2015, which was identified using National Death Index data. The causes of disease-specific mortality were defined using ICD-10. Participants who had ICD-10 codes of I00–I09, I11, I13, I20–I51, or I60–I69 were classified as having died from CVD. Meanwhile, those who had ICD-10 codes of C00–C97 were considered to have died from cancer. Briefly, in the analysis using DII as exposure, 1943 deaths were recorded, including 467 deaths from CVD and 470 deaths from cancer. And in the analysis using DOBS as exposure, 3616 deaths were observed, with 941 attributed to CVD and 819 to cancer.

### 2.7. Assessment of Covariates

The confounding variables included sex (male/female), age (years), race (Mexican American/other Hispanic/non-Hispanic White/non-Hispanic Black/other), BMI (kg/m^2^), cotinine (ng/mL), regular exercise (yes/no), education level (<9th grade/9–11th grade/high school graduate/GED or equivalent/college or Associate in Arts degree/college graduate or above), annual household income (<USD 20,000/USD 20,000–USD 45,000/USD 45,000–USD 75,000/USD 75,000–USD 100,000/>USD 100,000), total energy intake (kcal/day), dietary supplement use (yes/no), diet quality assessed using the Alternative Healthy Eating Index (AHEI) score [53], self-reported cancer (yes/no), CVD (yes/no), hypertension (yes/no), and diabetes (yes/no).

### 2.8. Cox Proportional Hazards (CPH) Regression Models

DII and DOBS were converted into categorical variables by quartiles and used in the corresponding analyses. CPH regression models were developed. The duration of survival was defined as the number of months between the NHANES interview date and either the date of death or the final census conducted on 31 December 2015. Moreover, the CPH regression models controlled for many potential effect modifiers and confounding factors, which were age, sex, race, body mass index (BMI), cotinine, regular exercise, education level, annual household income, total energy intake, dietary supplement use, alternative healthy eating index (AHEI), self-reported cancer, CVD, hypertension, and diabetes. To examine linear trends, the median DII/DOBS for each quantile was included as a continuous variable in the models.

### 2.9. Competing Risk Models

Inflammation and oxidative stress are important risk factors for CVD, cancer, and all other causes of death during the follow-up period prior to the onset of these two outcomes. Therefore, it is highly necessary to apply competing risk models to test the potential impact of competing events. As a special type of survival analysis, a competing risk model accurately estimates the marginal probability of an outcome of interest occurring independently of competing events [54,55]. Competing events were defined as any type of death except CVD/cancer. A multivariate competing risk model was performed to compare whether there were differences in the cumulative risk of outcome events in different DII/DOBS quantiles after adjusting for potential covariates and controlling for competing events. The adjusted covariates in this part of the analysis were kept the same as those in the CPH regression model.

### 2.10. Mediation Statistical Models

To investigate whether inflammatory markers could act as mediators in the relationship between DII/DOBS and mortality, mediation statistical models were constructed with adjustment for the above covariables. The process of designing and developing mediation statistical models has been described elsewhere [56].

### 2.11. Sensitivity Analysis

To verify the reliability of our findings, five sets of sensitivity analyses were additionally conducted as follows: (1) the association of the combination of DII and DOBS with mortality was investigated among the co-participants of the two analyses, in which participants were reclassified into those with pro-inflammatory and pro-oxidative diets (the third quantile of DII and the first quantile of DOBS), anti-inflammatory and antioxidant diets (the first quantile of DII and the third quantile of DOBS), and a composite diet (participants not in the above two groups). (2) After adjusting for potential covariates and competing events, a multivariate competing risk model was used to explore whether there was a difference in the cumulative risk of outcome events across different DII/DOBS quantiles. (3) Restricted cubic splines (RCS) were applied to visualize the linearity of the relationships between DII/DOBS and mortality. (4) Mediation statistical models were carried out to investigate the mechanisms underlying the relationships found in this study by assessing the impacts of mediator variables. (5) A series of subgroup analyses were performed to reveal how DII/DOBS was associated with mortality in specific populations.

### 2.12. Statistical Analyses

All analyses in this study took into account sample weights, as well as stratification and clustering methods, in order to meet the requirements of the NHANES analysis guidelines for complex sampling designs. Continuous and categorical demographic, anthropometric, and dietary intake variables are shown as means (95% CI) and percentages (95% CI), respectively. To compare continuous and categorical baseline characteristics, age-adjusted general linear models and χ^2^ tests were adopted, respectively.

All analyses mentioned above were performed using R 4.1.1, and statistically significant results were defined as *p* < 0.05.

## 3. Results

### 3.1. Baseline Characteristics

The differences in the demographic and nutritional characteristics according to DII/DOBS in quartiles are described in Supplementary Tables S3 and S4. Significant differences were observed in the study variables, except for annual household income, among the different DII/DOBS quartiles (*p* < 0.05). Participants eating pro-inflammatory and pro-oxidative diets were more likely to be Hispanic white and female and had higher cotinine, BMI, CRP, RDW, prevalent CVD, hypertension and diabetes. Additionally, they had lower education levels, socioeconomic status, dietary supplement use, total energy intake, AHEI, and cancer incidence (Table 1).

### 3.2. Multivariate Linear Regression Models

In the validation study, there were strong positive correlations of DII with serum CRP (β 0.08, 95% CI 0.04–0.13) and RDW (β 0.18, 95% CI 0.11–0.24). DOBS was inversely correlated with serum CRP (β −0.13, 95% CI −0.18–−0.09) and RDW (β −0.10, 95% CI −0.17–−0.02) (Supplementary Tables S5 and S6).

### 3.3. CPH Regression Models

**Figure 2:**
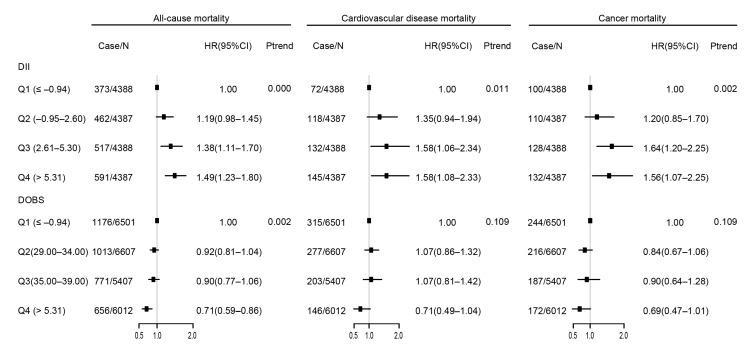
Association of DII and DOBS with mortality. Data are presented as the weighted hazard ratio (HR) estimates and 95% confidence intervals. The adjustments include age, sex, ethnicity, BMI, cotinine, exercise, education, income, nutrient supplement use, AHEI, self-reported cancer, self-reported cardiovascular disease, self-reported hypertension, and self-reported diabetes. Case/N, the number of case subjects/total. Q, quintile.

**Figure 3:**
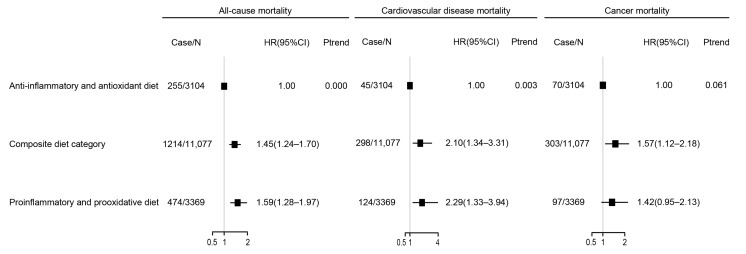
Association of the combination of DII and DOBS with mortality. Data are presented as weighted HR estimates and 95% confidence intervals. The adjustments include age, sex, ethnicity, BMI, cotinine, exercise, education, income, nutrient supplement use, AHEI, self-reported cancer, self-reported cardiovascular disease, self-reported hypertension, and self-reported diabetes. Case/N, the number of case subjects/total. Q, quintile.

The relationship between DII/DOBS and mortality is presented in Figure 2 and Supplementary Tables S7 and S8. Participants in the highest quartile of DII (quartile 4) were more likely to die due to all-cause diseases (HR 1.49, 95% CI 1.23–1.80), CVD (HR 1.58, 95% CI 1.08–2.33), and cancer (HR 1.56, 95% CI 1.07–2.25). For DOBS, participants in quartile 4 had significantly lower all-cause mortality (HR 0.71, 95% CI 0.59–0.86) compared with those in quartile 1. Meanwhile, DOBS was not significantly associated with CVD (HR 0.71, 95% CI 0.49–1.04) or cancer mortality (HR 0.69, 95% CI 0.47–1.01). The associations of the combination of DII and DOBS with risk of mortality are shown in Figure 3 and Supplementary Table S9. Pro-inflammatory and pro-oxidative diets were significantly related to all-cause (HR 1.59, 95% CI 1.28–1.97) and CVD (HR 2.29, 95% CI 1.33–3.94) mortality.

### 3.4. Restricted Cubic Splines (RCS)

**Figure 4:**
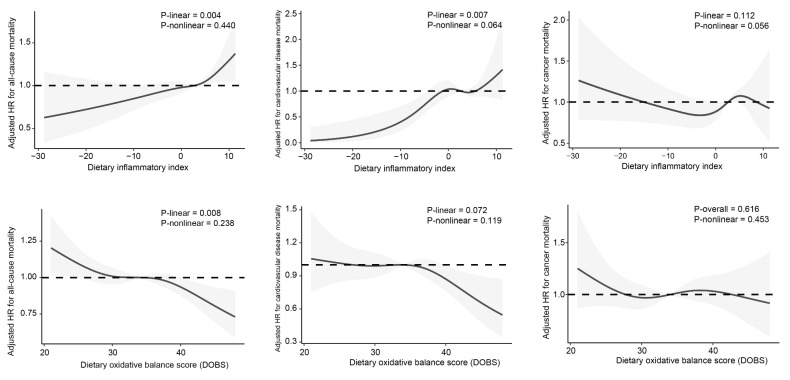
Associations of DII and DOBS with mortality, evaluated using CPH regression models and RCS after adjusting for age, sex, ethnicity, BMI, cotinine, exercise, education, income, nutrient supplement use, AHEI, self-reported cancer, self-reported cardiovascular disease, self-reported hypertension, and self-reported diabetes. Central estimates are represented by solid black lines, and gray shaded areas indicate 95% confidence intervals.

The association between DII/DOBS and mortality was modeled flexibly using RCS, and the results are illustrated in Figure 4. DII had approximate linear associations with a higher risk of all-cause (P_linearity_ = 0.004, P_nonlinearity_ = 0.440) and CVD death (P_linearity_ = 0.007, P_nonlinearity_ = 0.064) after multivariable adjustment. Similarly, a significantly opposite trend was detected between DOBS and all-cause mortality (P_linearity_ = 0.008, P_nonlinearity_ = 0.238).

### 3.5. Sensitivity Analysis

Supplementary Tables S10–S12 show the results of competing risk models after controlling for competing risk events. The results reveal consistently similar association patterns between DII, the combination of DII and DOBS, and CVD/cancer mortality. Both DII (HR 1.44, 95% CI 1.05–1.98) and a pro-inflammatory and pro-oxidative diets were independent factors of higher CVD death (HR 1.83, 95% CI 1.26–2.65). According to the association found in the study, statistical mediation models were established. The mediation effects are shown in Supplementary Figure S1. The total effect of DII on all-cause (β_Tot_ = 0.00335, *p* < 0.01) and CVD (β_Tot_ = 0.00115, *p* < 0.001) death was measured as a standardized regression coefficient. The total effect of DOBS on all-cause mortality (β_Tot_ = −0.00222, *p* < 0.01) also confirmed the previous findings. The total effect of the combination of DII and DOBS on all-cause (β_Tot_ = 0.02189, *p* < 0.01) and CVD (β_Tot_ = 0.01303, *p* < 0.001) mortality was measured after adjusting for the corresponding covariables in the CPH regression models. The indirect effect was calculated using β1, β2, β3, and β4. The proportions of the indirect effect on all-cause and CVD mortality mediated by CRP and RDW were measured at 5.97, 8.70, 4.48, 4.35%, respectively. The indirect effect mediated by CRP (4.05%) and RDW (5.86%) also accounted for a certain percentage of the total effect of DOBS on all-cause death. The proportions of the indirect effect on all-cause and CVD mortality mediated by CRP and RDW were 4.66, 3.68, 8.54, 4.22%, respectively. Our results were not materially changed during most stratification analyses according to age, sex, race, BMI, smoking, and exercise status (Supplementary Tables S13–S21). Especially in the participants who were aged 45 years or younger, were male, were non-Hispanic white, did not smoke, and performed regular exercise, the results did not change significantly. Furthermore, BMI had no modification effect on the above associations (Supplementary Tables S13–S21).

## 4. Discussion

Using 26 dietary components associated with inflammatory potential and 17 a priori selected pro- and antioxidant nutrients, we constructed two holistic scores that comprehensively represent an individual’s dietary pro-inflammatory and antioxidant levels. Our results confirmed the findings of previous studies, indicating that higher DII or lower DOBS was associated with increased all-cause mortality [31,49]. This study found that people who consumed more inflammatory foods showed an increased risk of CVD and cancer death. Furthermore, consuming more both pro-inflammatory and pro-oxidative foods was related to greater all-cause and CVD mortality. Last, but not least, younger participants, men, non-Hispanic white participants, non-smokers, and those who performed regular exercise experienced a reduced risk of death when they adhered to a diet rich in anti-inflammatory or antioxidant properties.

To the best of our knowledge, this study was the first to examine the association of dietary-derived anti-inflammatory and/or antioxidant capacity with mortality. Chronic low-level inflammatory responses and oxidative stress are important in the occurrence of chronic diseases and death [57,58]. As important physiological processes in the body, they are also affected by many factors, including genetics, age, diet, lifestyle, immunity, and environment [59]. In line with previous studies, we confirmed that higher DII or lower DOBS was associated with the increased levels CRP and RDW, which were more strongly related to disease, aging, and death [60,61,62,63]. This discovery indirectly supports the association of DII/DOBS with poor health.

Several published meta-analyses have confirmed the role of dietary-induced inflammation [31,64,65,66]. Adherence to pro-inflammatory dietary patterns was significantly and positively associated with poor health status in more than half of the included studies. Consistent with our findings, Class I (Convincing) evidence was established only for CVD and CVD-related death according to the results of the credibility assessment grade division. The evidence of increased all-cause mortality and overall and site-specific cancer incidence with increasing DII was determined to be Class II (highly suggestive). However, other outcomes had Class III (suggestive) evidence [67]. In the absence of interventional studies aimed at modifying the inflammatory potential of the diet and examining its effects on the above-mentioned outcomes of risk factors and diseases, it was impossible to determine whether DII functioned independently of body composition. Considering the observed correlation between DII and systemic inflammatory biomarkers like CRP and RDW, it was reasonable to hypothesize that systemic inflammation might serve as a mediator between DII and mortality [68]. Our mediation analysis suggested that CRP and RDW partly mediated the association of DII with the risk of death, despite the fact that the effect was relatively small in magnitude.

Due to the great heterogeneity in the definition of oxidative balance scores (OBSs), different scoring schemes and various kinds of pro- and antioxidant components were presented [48]. Most OBSs were derived by considering more than 10 components (dietary intake and lifestyles), and few considered only dietary factors [48]. Most studies were consistent in supporting that excessive oxidative stress reflected by lower OBSs had deleterious effects on health. There is limited research available on the potential link between DOBS and mortality. Among a biracial US cohort, individuals who consumed a diet with greater antioxidant capacity had lower mortality [49]. It was unclear whether the different results of the association of DOBS with the risk of CVD and cancer death could be attributed to variations in designs or differences in the characteristics of the populations being studied. In addition, lower DOBS has been associated with the elevated levels of CRP and RDW, which were more strongly linked to death and mediated the association.

Estimates for the risk of death based on combining DII and DOBS were stronger, especially for all-cause and CVD mortality. They were was also consistent with our previous findings that when individuals had either higher DII or lower DOBS alone, they still faced a higher likelihood of mortality. Compared to all-cause and CVD mortality, cancer death was more weakly associated with the levels of components in the diet related to inflammation and oxidative stress. This may be due to the fact that the mechanisms of many cancers have little or no relationship with inflammation and oxidative stress, such as genetic mutations, the inactivation of tumor suppressor genes, and matrix degradation [69], which have been considered major drivers of tumor development. This had been reported in many previous studies, as well [40,70]. Confusingly, the baseline characteristics showed that participants eating pro-inflammatory and pro-oxidative diets had lower cancer incidence. This phenomenon may not be verified, and may be due to the facts that the self-reported cancer-related data came from questionnaires, the exact time of cancer diagnoses were unknown, and the self-reported cancer prevalence may not match current dietary intake information [42,43].

In the sensitivity analysis of our study, similar associations were observed when DII, DOBS, and the combination of DII and DOBS were analyzed separately. In addition, stronger associations were observed among participants who were aged 45 years or younger, were male, were non-Hispanic white, did not smoke, and performed regular exercise, which was aligned with the data from the *Reasons for Geographic and Racial Differences in Stroke* (REGARDS) Study [49]. One possible explanation for this could be the healthier lifestyles, lower disease risk, and physiological and metabolic differences in these individuals, so the relationship between diet quality and health risks may be more pronounced.

The present study incorporated several notable strengths. Firstly, this was the first study to examine the association of dietary inflammation and oxidative stress (the combination of DII and DOBS) with all-cause, CVD, and cancer mortality based on rigorously designed population-based research conducted using NHANES. Second, the use of a complex, multistage probability sampling design ensured that the sample in this study was representative of non-institutionalized civilian residents, which allowed the findings to be applicable to all adult residents who were not institutionalized across the United States. Meanwhile, the association was proven to be fairly robust with adjustment for various important confounders, and still very robust in the sensitivity analysis. However, this study also had certain limitations. First, using DII and DOBS, it was hard to include all instances of inflammatory response- and oxidative stress-associated dietary exposure; many components, such as isoflavones, anthocyanidins, and tea, were limited in the database. Some nutrients that have been found to be associated with inflammation and oxidative stress but have not yet been demonstrated, such as calcium, were also not included. However, our study incorporated as many relevant components as possible compared to similar published studies. And the association between current DII/DOBS and mortality was sufficiently stable and unlikely to be substantially impacted by the elements that were not incorporated. Second, dietary information was gathered from the self-reported 24 h dietary recall, and it cannot be excluded that the participants might have altered their dietary patterns. Third, this study directly assumed that oxidative stress was linearly correlated with both pro-oxidants and antioxidants, without taking into account the potential threshold effect. Nonetheless, research studies have discovered that certain antioxidants, such as carotenoids and copper, may actually demonstrate pro-oxidative activity when administered at high doses or under specific conditions [71]. Fourth, no well-matched verifiable markers of oxidative stress were included for evaluating the effectiveness of DOBS. Finally, despite the inclusion of several confounding variables, there was still a high probability that certain confounders were not taken into account.

## 5. Conclusions

The results from this nationally representative sample suggested that lower DII or higher DOBS may reduce the risk of premature all-cause and CVD death. Participants whose diets were both anti-inflammatory and antioxidant were at lower risk of all-cause and CVD mortality, especially those who were aged 45 years or younger, were male, were non-Hispanic white, did not smoke, and performed regular exercise. Cancer death was only weakly associated with the levels of components in the diet related to inflammation. These findings confirm the results of previous research and suggested that DII and DOBS might be valuable instruments for assessing the impacts of dietary factors associated with inflammation and oxidative stress on morbidity and mortality. To understand the exact underlying mechanisms, further investigation is needed.

## Figures and Tables

**Table 1:** Baseline characteristics according to the different combinations of DII and DOBS ^a^.

	The Different Combinations of DII and DOBS (*N* = 17,750)			
	Anti-Inflammatory and Antioxidant Diet	Composite Diet	Proinflammatory and Pro-oxidative Diet	
	*N* = 3104	*N* = 11,077	*N* = 3369	*p*
Age, years	47.11 (46.16, 48.05)	47.16 (46.55, 47.77)	47.14 (46.29, 47.99)	0.987
Male (%)	67.70 (65.90, 69.50)	46.50 (45.30, 47.60)	29.00 (26.80, 31.40)	0.000
Non-Hispanic white (%)	73.90 (70.70, 76.90)	69.60 (66.10, 72.80)	62.30 (56.70, 67.50)	0.000
BMI, kg/m^2^	28.04 (27.69, 28.39)	28.93 (28.75, 29.10)	29.64 (29.36, 29.92)	0.000
Cotinine (ng/mL)	34.17 (28.90, 39.44)	54.09 (49.23, 58.95)	86.22 (77.84, 94.60)	0.000
Regular exercise (%)	26.10 (23.70, 28.80)	19.50 (18.00, 21.10)	16.90 (15.00, 18.90)	0.000
College graduate or above (%)	42.90 (40.00, 45.90)	30.40 (28.30, 32.60)	13.10 (11.00, 15.50)	0.000
>USD 100,000 annual household income (%)	31.60 (28.00, 35.50)	23.10 (21.00, 25.40)	13.40 (11.20, 15.80)	0.000
Dietary supplement use (%)	60.90 (58.10, 63.60)	52.80 (50.90, 54.60)	40.30 (38.00, 42.60)	0.000
Total energy, kcal/day	2683 (2652, 2713)	2036 (2017, 2055)	1400 (1375, 1425)	0.000
AHEI sore	52.94 (52.35, 53.52)	49.71 (49.37, 50.04)	48.24 (47.84, 48.63)	0.000
Self-reported cancer (%)	10.10 (9.00, 11.40)	9.40 (8.60, 10.20)	9.60 (8.20, 11.20)	0.000
Self-reported cardiovascular disease (%)	6.60 (5.60, 7.80)	7.70 (7.10, 8.40)	11.20 (9.70, 13.00)	0.000
Self-reported hypertension (%)	28.60 (26.40, 31.00)	31.40 (29.90, 33.00)	34.20 (32.20, 36.30)	0.000
Self-reported diabetes (%)	7.40 (6.20, 8.70)	8.80 (8.00, 9.60)	11.30 (10.20, 12.50)	0.000
C-reaction protein (mg/dL)	0.29 (0.25, 0.33)	0.38 (0.36, 0.40)	0.48 (0.43, 0.52)	0.000
Red cell distribution width (%)	12.80 (12.75, 12.85)	12.99 (12.94, 13.04)	13.24 (13.18, 13.30)	0.000

^a^ Continuous variables are listed as weighted means (95% CI). Categorical variables are listed as weighted percentages (95% CI). After adjusting for age, general linear models and chi-square tests were conducted to compare continuous and categorical baseline characteristics, respectively.

## Data Availability

The data utilized are openly accessible, as mentioned earlier.

## Supplementary Materials

The following supporting information can be downloaded at: https://www.mdpi.com/article/10.3390/nu15143148/s1, Table S1. Dietary composition parameters involved in DII. Table S2. The DOBS assignment scheme. Table S3. Baseline characteristics according to DII quartiles. Table S4. Baseline characteristics according to DOBS quartiles. Table S5. Association of DII with serum CRP and RDW. Table S6. Association of DOBS with CRP and RDW. Table S7. Association of DII with mortality. Table S8. Association of DOBS with mortality. Table S9. Association of the combination of DII and DOBS with mortality. Table S10. Association of DII with mortality in competing risk models. Table S11. Association of DOBS with mortality in competing risk models. Table S12. Association of the combination of DII and DOBS with mortality in competing risk models. Table S13. Association of DII with all-cause mortality stratified by age, sex, race, BMI, smoking, and exercise status. Table S14. Association of DII with CVD mortality stratified by age, sex, race, BMI, smoking, and exercise status. Table S15. Association of DII with cancer mortality stratified by sex, race, BMI, smoking, and exercise status. Table S16. Association of DOBS with all-cause mortality stratified by age, sex, race, BMI, smoking, and exercise status. Table S17. Association of DOBS with CVD mortality stratified by age, sex, race, BMI, smoking, and exercise status. Table S18. Association of DOBS with cancer mortality stratified by sex, race, BMI, smoking, and exercise status. Table S19. Association of the combination of DII and DOBS with all-cause mortality stratified by age, sex, race, BMI, smoking, and exercise status. Table S20. Association of the combination of DII and DOBS with CVD mortality stratified by age, sex, race, BMI, smoking, and exercise status. Table S21. Association of the combination of DII and DOBS with cancer mortality stratified by sex, race, BMI, smoking, and exercise status. Figure S1. Mediation effects of CRP and RDW on the associations of DII/DOBS/the combination of DII and DOBS with all-cause, CVD, and cancer mortality.
