# Supplementary material for: Association of Dietary Inflammatory Index and Dietary Oxidative Balance Score with All-Cause and Disease-Specific Mortality: Findings of 2003–2014 National Health and Nutrition Examination Survey
Source: Nutrients. 2023 Jul 14;15(14):3148. doi: 10.3390/nu15143148 (PMC10383761; doi:10.3390/nu15143148)

Supplementary table S1. Dietary composition parameters involved in DII, inflammatory effect scores, and intake values from the global composite data set[d]

Dietary composition parameter	Raw inflammatory effect score[b]	Overall inflammatory effect score[c]	Global daily mean intake (units/d)	Standard deviation of the global daily intake
Alcohol (g)	-0.278	-0.278	13.98	3.72
Vitamin B12 (μg)	0.205	0.106	5.15	2.7
Vitamin B6 (mg)	-0.379	-0.365	1.47	0.74
β-Carotene (μg)	-0.584	-0.584	3718	1720
Caffeine (g)	-0.124	-0.110	8.05	6.67
Carbohydrate (g)	0.109	0.097	272.2	40
Cholesterol (mg)	0.347	0.110	279.4	51.2
Energy (kcal)	0.180	0.180	2056	338
Total fat (g)	0.298	0.298	71.4	19.4
Fiber (g)	-0.663	-0.663	18.8	4.9
Folic acid (μg)	-0.207	-0.190	273	70.7
Iron (mg)	0.032	0.032	13.35	3.71
Magnesium (mg)	-0.484	-0.484	310.1	139.4
MUFA[e] (g)	-0.019	-0.009	27	6.1
Niacin (mg)	-1.000	-0.246	25.9	11.77
Protein (g)	0.049	0.021	79.4	13.9
PUFA[f] (g)	-0.337	-0.337	13.88	3.76
Vitamin B2 (mg)	-0.727	-0.068	1.7	0.79
Saturated fat (g)	0.429	0.373	28.6	8
Selenium (μg)	-0.191	-0.191	67	25.1
Vitamin B1 (mg)	-0.354	-0.098	1.7	0.66
Vitamin A (RE[a])	-0.401	-0.401	983.9	518.6
Vitamin C (mg)	-0.424	-0.424	118.2	43.46
Vitamin D (μg)	-0.446	-0.446	6.26	2.21
Vitamin E (mg)	-0.419	-0.419	8.73	1.49

Zinc (mg)	-0.313	-0.313	9.84	2.19

[a]Retinol equivalents.

[b]Dietary composition parameter-specific raw inflammatory effect score, which is per unit amount noted for each food parameter.

[c]Dietary composition parameter-specific overall inflammatory effect score.

[d]DII of a certain dietary component = (Daily intake of the dietary component - Global daily mean intake of the dietary component) / Standard deviation of the global daily intake for the dietary component * Overall inflammatory effect score of the dietary component. The DII for each participant was obtained by summing the DII of the 26 dietary components selected in this study.

[e]Monounsaturated fatty acids.

[f]Polyunsaturated fatty acids.

Supplementary table S2. The DOBS assignment scheme

DOBS components	Property	DOBS score		
		1	2	3
Dietary fiber (g/d)	A[a]	≤12.50	12.51-18.75	＞18.76
Ln-transformed carotene (μg/d)	A	≤8.31	8.32-9.20	＞9.21
Vitamin B2 (mg/d)	A	≤1.57	1.58-2.46	＞2.47
Niacin (mg/d)	A	≤18.30	18.31-26.75	＞26.76
Vitamin B6 (mg/d)	A	≤1.46	1.47-2.38	＞2.39
Folic acid (μg/d)	A	≤288.00	288.01-430.50	＞430.51
Ln-transformed vitamin B12 (μg/d)	A	≤0.94	0.95-1.21	＞1.22
Ln-transformed vitamin C (mg/d)	A	≤3.88	3.89-4.67	＞4.68
Vitamin E (mg/d)	A	≤5.81	5.82-9.25	＞9.26
Calcium (mg/d)	A	≤643.00	643.01-994.50	＞994.51
Magnesium (mg/d)	A	≤224.00	224.01-314.00	＞314.01
Zinc (mg/d)	A	≤9.06	9.07-12.86	＞12.87
Copper (mg/d)	A	≤0.99	1.00-1.47	＞1.48
Selenium (μg/d)	A	≤83.60	83.61-120.90	＞120.91
Total fat (g/d)	P[b]	＞84.94	56.55-84.93	≤56.54
Iron (mg/d)	P	＞16.53	11.44-16.52	≤11.43
Alcohol (male) (g/d)	P	＞30.01	0.01-30.00	0.00
Alcohol (female) (g/d)	P	＞15.01	0.01-15.00	0.00

[a]Antioxidant.

[b]Prooxidant.

Supplementary table S3. Baseline characteristics according to dietary inflammatory index (DII) quartiles[a]

	Dietary inflammatory index (DII) (N = 17,750)				
	≤ - 0.94<br>N = 4,388	-0.95 - 2.60<br>N = 4,387	2.61 - 5.30<br>N = 4,388	> 5.31<br>N = 4,387	*P*
Age, years	47.50 (46.63,48.36)	47.62 (46.85,48.38)	47.10 (46.22,47.79)	46.28 (45.57,46.98)	0.034
Male, (%)	62.40 (60.70,64.10)	50.30 (48.50,52.00)	41.30 (39.40,43.20)	33.40 (31.70,35.20)	0.000
Non-Hispanic white (%)	74.50 (71.50,77.30)	70.50 (67.10,73.60)	66.10 (61.70,70.20)	64.50 (59.20,69.40)	0.013
BMI, kg/m^2	27.92 (27.60,28.24)	28.60 (28.37,28.84)	29.50 (29.17,29.83)	29.71 (29.46,29.96)	0.000
Cotinine (ng/ml)	37.72 (32.95,42.48)	47.10 (40.71,53.49)	54.69 (48.29,61.08)	88.50 (80.73,96.27)	0.000
Regular exercise (%)	25.70 (23.20,28.30)	21.40 (19.20,23.80)	17.00 (15.00,19.10)	16.10 (14.50,17.90)	0.000
College graduate or above (%)	42.50 (39.70,45.40)	34.90 (32.30,37.50)	24.40 (22.30,26.60)	14.60 (12.50,16.90)	0.000
> 100,000 annual household income (%)	32.00 (28.60,35.60)	25.30 (22.80,28.00)	19.50 (17.50,21.70)	13.40 (11.40,15.80)	0.319
Dietary supplements use (%)	60.60 (58.20,62.90)	54.90 (52.80,57.00)	50.20 (48.10,52.30)	41.20 (38.90,43.40)	0.000
Total energy, kcal/day	2527 (2497,2556)	2147 (2122,2172)	1885 (1858,1912)	1556 (1531,1582)	0.000
AHEI sore	52.54 (52.06,53.02)	50.51 (50.11,50.91)	49.06 (48.58,49.54)	47.64 (47.27,48.00)	0.000
Self-reported cancer (%)	9.90 (9.00,11.00)	9.70 (8.60,10.80)	9.20 (8.00,10.50)	9.40 (8.20,10.90)	0.000
Self-reported cardiovascular diseases (%)	6.20 (5.30,7.10)	7.10 (6.10,8.20)	9.30 (8.00,10.80)	10.30 (9.20,11.60)	0.000
Self-reported hypertension (%)	29.00 (26.70,31.30)	30.50 (28.30,32.80)	32.90 (31.20,34.60)	33.60 (32.00,35.30)	0.000
Self-reported diabetes (%)	6.90 (5.80,8.10)	8.80 (7.60,10.30)	10.20 (9.00,11.50)	10.20 (9.30,11.20)	0.000
C reaction protein (mg/dL)	0.28 (0.24,0.32)	0.35 (0.33,0.38)	0.42 (0.39,0.46)	0.48 (0.44,0.51)	0.000
Red cell distribution width (%)	12.82 (12.78,12.87)	12.96 (12.90,13.03)	13.05 (12.99,13.12)	13.18 (13.13,13.23)	0.000

[a]Continuous variables were listed as weighted mean (95% CI). Categorical variables were listed as weighted percentage (95% CI). After adjusting for age, general linear models and chi-square tests were conducted to compare continuous and categorical baseline characteristics, respectively.

Supplementary table S4. Baseline characteristics according to dietary oxidative balance score (DOBS) quartiles: NHANES, 2003–2014[a]

	Dietary oxidative balance score (DOBS) (N = 24,527)				
	≤ 28.00<br>N = 6,501	29.00 - 34.00<br>N = 6,607	35.00 - 39.00<br>N = 5,407	> 40.00<br>N = 6,012	*P*
Age, years	47.76 (47.18,48.34)	47.46 (46.84,48.08)	47.40 (46.72,48.08)	46.36 (45.62,47.09)	0.005
Male, (%)	29.30 (27.90,30.80)	40.90 (39.40,42.40)	51.30 (49.80,52.90)	67.50 (66.00,68.90)	0.000
Non-Hispanic white (%)	64.40 (60.70,68.00)	69.10 (65.80,72.20)	72.90 (70.20,75.50)	75.90 (73.50,78.20)	0.000
BMI, kg/m^2	29.03 (28.81,29.25)	29.05 (28.86,29.24)	28.54 (28.28,28.80)	28.35 (28.10,28.60)	0.000
Cotinine (ng/ml)	80.71 (74.67,86.76)	59.40 (53.53,65.27)	54.16 (49.56,58.76)	41.59 (37.30,45.88)	0.000
Regular exercise (%)	23.90 (22.10,25.80)	25.40 (23.40,27.40)	26.80 (24.80,29.00)	31.60 (29.70,33.50)	0.000
College graduate or above (%)	16.60 (14.90,18.50)	26.00 (24.00,28.10)	32.50 (30.30,34.80)	39.00 (36.80,41.20)	0.000
> 100,000 annual household income (%)	10.20 (8.80,11.80)	14.80 (13.10,16.80)	17.60 (15.70,19.70)	20.40 (18.10,23.00)	0.226
Dietary supplements use (%)	45.70 (44.00,47.30)	52.90 (51.20,54.70)	55.40 (53.50,57.40)	58.60 (56.50,60.60)	0.000
Total energy, kcal/day	1456 (1440,1472)	1848 (1829,1866)	2208 (2187,2229)	2694 (2672,2716)	0.000
AHEI sore	48.64 (48.34,48.94)	49.57 (49.19,49.95)	50.08 (49.65,50.51)	51.80 (51.38,52.22)	0.000
Self-reported cancer (%)	9.00 (8.00,10.00)	9.60 (8.60,10.60)	9.80 (8.80,10.80)	8.90 (8.10,9.80)	0.000
Self-reported cardiovascular diseases (%)	11.00 (10.00,12.10)	8.80 (7.90,9.70)	7.90 (7.10,8.80)	6.30 (5.50,7.10)	0.000
Self-reported hypertension (%)	34.30 (32.90,35.60)	32.50 (31.00,33.90)	30.30 (28.40,32.30)	28.30 (26.50,30.30)	0.000
Self-reported diabetes (%)	9.90 (9.10,10.70)	9.10 (8.10,10.20)	8.10 (7.20,9.10)	7.40 (6.60,8.20)	0.000
C reaction protein (mg/dL)	0.48 (0.45,0.51)	0.44 (0.41,0.48)	0.37 (0.34,0.39)	0.31 (0.29,0.34)	0.000
Red cell distribution width (%)	13.08 (13.04,13.12)	12.95 (12.90,13.00)	12.85 (12.81,12.89)	12.74 (12.69,12.78)	0.000

[a]Continuous variables were listed as weighted mean (95% CI). Categorical variables were listed as weighted percentage (95% CI). After adjusting for age, general linear models and chi-square tests were conducted to compare continuous and categorical baseline characteristics, respectively.

Supplementary table S5. Association of the DII with serum C-reaction protein (CRP) and red cell distribution width (RDW)

		DII[c]							
		Q1[d]	Q2		Q3		Q4		P_{trend}
			β[b]	95% CI	β	95% CI	β	95% CI	
CRP									
	Model 1[a]	Ref (0)[e]	0.06	**(0.02,0.10)****	0.13	**(0.07,0.18)*****	0.18	**(0.12,0.23)*****	0.000[f]
	Model 2	Ref (0)	0.04	(-0.01,0.08)	0.07	**(0.03,0.12)****	0.10	**(0.05,0.14)*****	0.000
	Model 3	Ref (0)	0.03	(-0.01,0.08)	0.07	**(0.02,0.12)***	0.08	**(0.04,0.13)****	0.001
RDW									
	Model 1	Ref (0)	0.12	**(0.06,0.18)*****	0.20	**(0.13,0.26)*****	0.32	**(0.25,0.38)*****	0.000
	Model 2	Ref (0)	0.09	**(0.03,0.15)****	0.12	**(0.06,0.19)*****	0.20	**(0.14,0.26)*****	0.000
	Model 3	Ref (0)	0.08	**(0.02,0.14)***	0.10	**(0.03,0.17)****	0.18	**(0.11,0.24)*****	0.000

[a]Model 1 was adjusted for age, sex, and ethnicity. Model 2 further adjusted for BMI, cotinine, exercise, education, and income. Model 3 further adjusted for nutrient supplement use, AHEI, self-reported cancer, self-reported cardiovascular diseases, self-reported hypertension, and self-reported diabetes.

[b]Data were listed as the weighted beta estimates and 95% confidence intervals calculated using generalized linear regression models, with *p < 0.05, **p < 0.01, ***p < 0.001.

[c]Higher DII indicates a predominance of proinflammatory exposure.

[d]Q, quintile.

[e]Ref, reference.

[f]Tests for trends based on the variables containing the median values for each quartile.

Supplementary table S6. Association of the DOBS with serum C-reaction protein (CRP) and red cell distribution width (RDW)

		DOBS[c]							
		Q1[d]	Q2		Q3		Q4		P_{trend}
			β[b]	95% CI	β	95% CI	β	95% CI	
CRP									
	Model 1[a]	Ref (0)[e]	-0.03	(-0.08,0.02)	-0.09	**(-0.13,-0.06)***	-0.13	**(-0.18,-0.09)***	0.000[f]
	Model 2	Ref (0)	-0.02	(-0.06,0.03)	-0.07	**(-0.11,-0.03)***	-0.10	**(-0.14,-0.06)***	0.000
	Model 3	Ref (0)	-0.01	(-0.05,0.04)	-0.05	**(-0.09,-0.01)***	-0.06	**(-0.11,-0.01)***	0.007
RDW									
	Model 1	Ref (0)	-0.11	**(-0.17,-0.05)***	-0.19	**(-0.24,-0.13)***	-0.26	**(-0.32,-0.21)***	0.000
	Model 2	Ref (0)	-0.08	**(-0.14,-0.03)**	-0.13	**(-0.18,-0.08)***	-0.18	**(-0.23,-0.13)***	0.000
	Model 3	Ref (0)	-0.05	(-0.11,0.00)	-0.08	**(-0.14,-0.02)***	-0.10	**(-0.17,-0.02)***	0.013

[a]Model 1 was adjusted for age, sex, and ethnicity. Model 2 further adjusted for BMI, cotinine, exercise, education, and income. Model 3 further adjusted for total intake of energy, nutrient supplement use, AHEI, self-reported cancer, self-reported cardiovascular diseases, self-reported hypertension, and self-reported diabetes.

[b]Data were listed as the weighted beta estimates and 95% confidence intervals calculated using generalized linear regression models, with *p < 0.05, **p < 0.01, ***p < 0.001.

[c]Higher DOBS indicates a predominance of antioxidant exposure.

[d]Q, quintile.

[e]Ref, reference.

[f]Tests for trends based on the variables containing the median values for each quartile.

Supplementary table S7. Association of DII with mortality

		DII							
		Q1[c]	Q2		Q3		Q4		P_{trend}
			HR[b]	95% CI	HR	95% CI	HR	95% CI	
All-cause mortality									
	Model 1[a]	Ref (1)[d]	1.29	**(1.06,1.58)***	1.70	**(1.42,2.05)*****	1.95	**(1.63,2.33)*****	0.000[e]
	Model 2	Ref (1)	1.22	(1.00,1.48)	1.48	**(1.21,1.80)*****	1.60	**(1.34,1.92)*****	0.000
	Model 3	Ref (1)	1.19	(0.98,1.45)	1.38	**(1.11,1.70)****	1.49	**(1.23,1.80)*****	0.000
CVD mortality									
	Model 1	Ref (1)	1.56	**(1.07,2.28)***	2.25	**(1.52,3.31)*****	2.35	**(1.58,3.50)*****	0.000
	Model 2	Ref (1)	1.40	(0.97,2.04)	1.78	**(1.21,2.60)****	1.78	**(1.21,2.60)****	0.001
	Model 3	Ref (1)	1.35	(0.94,1.94)	1.58	**(1.06,2.34)***	1.58	**(1.08,2.33)***	0.011
Cancer mortality									
	Model 1	Ref (1)	1.20	(0.85,1.70)	1.69	**(1.26,2.27)****	1.63	**(1.14,2.32)****	0.001
	Model 2	Ref (1)	1.19	(0.84,1.68)	1.59	**(1.16,2.19)****	1.52	**(1.07,2.14)***	0.002
	Model 3	Ref (1)	1.20	(0.85,1.70)	1.64	**(1.20,2.25)****	1.56	**(1.07,2.25)***	0.002

[a]Model 1 was adjusted for age, sex, and ethnicity. Model 2 further adjusted for BMI, cotinine, exercise, education, and income. Model 3 further adjusted for nutrient supplement use, AHEI, self-reported cancer, self-reported cardiovascular diseases, self-reported hypertension, and self-reported diabetes.

[b]Data were listed as the weighted hazard ratio estimates and 95% confidence intervals calculated using cox proportional hazards regression models, with *p < 0.05, **p < 0.01, ***p < 0.001.

[c]Q, quintile.

[d]Ref, reference.

[e]Tests for trends based on the variables containing the median values for each quartile.

Supplementary table S8. Association of DOBS with mortality

		DOBS							
		Q1[c]	Q2		Q3		Q4		P_{trend}
			HR[b]	95% CI	HR	95% CI	HR	95% CI	
All-cause mortality									
	Model 1[a]	Ref (1)[d]	0.83	**(0.74,0.92)****	0.77	**(0.68,0.87)*****	0.55	**(0.48,0.62)*****	0.000[e]
	Model 2	Ref (1)	0.90	(0.81,1.00)	0.87	**(0.77,0.98)***	0.66	**(0.59,0.74)*****	0.000
	Model 3	Ref (1)	0.92	(0.81,1.04)	0.90	(0.77,1.06)	0.71	**(0.59,0.86)*****	0.002
CVD mortality									
	Model 1	Ref (1)	0.91	(0.76,1.10)	0.81	**(0.67,0.98)***	0.45	**(0.35,0.57)*****	0.000
	Model 2	Ref (1)	0.99	(0.82,1.19)	0.92	(0.76,1.12)	0.55	**(0.44,0.70)*****	0.000
	Model 3	Ref (1)	1.07	(0.86,1.32)	1.07	(0.81,1.42)	0.71	(0.49,1.04)	0.207
Cancer mortality									
	Model 1	Ref (1)	0.80	**(0.65,0.98)***	0.86	(0.65,1.15)	0.49	**(0.49,0.80)*****	0.002
	Model 2	Ref (1)	0.86	(0.70,1.07)	0.95	(0.72,1.26)	0.74	**(0.57,0.95)***	0.062
	Model 3	Ref (1)	0.84	(0.67,1.06)	0.90	(0.64,1.28)	0.69	(0.47,1.01)	0.109

[a]Model 1 was adjusted for age, sex, and ethnicity. Model 2 further adjusted for BMI, cotinine, exercise, education, and income. Model 3 further adjusted for total intake of energy, nutrient supplement use, AHEI, self-reported cancer, self-reported cardiovascular diseases, self-reported hypertension, and self-reported diabetes.

[b]Data were listed as the weighted hazard ratio estimates and 95% confidence intervals calculated using cox proportional hazards regression models, with *p < 0.05, **p < 0.01, ***p < 0.001.

[c]Q, quintile.

[d]Ref, reference.

[e]Tests for trends based on the variables containing the median values for each quartile.

Supplementary table S9. Association of the combination of DII and DOBS with all-cause, cardiovascular disease (CVD), and cancer mortality

		The combination of DII and DOBS					P_{trend}
		Anti-inflammatory and antioxidant diet	Composite diet category		Proinflammatory and prooxidative diet		
			HR[b]	95% CI	HR	95% CI	
All-cause mortality							
	Model 1[a]	Ref (1)[c]	1.67	**(1.43,1.95)*****	2.15	**(1.73,2.67)*****	0.000[d]
	Model 2	Ref (1)	1.49	**(1.28,1.75)*****	1.72	**(1.40,2.12)*****	0.000
	Model 3	Ref (1)	1.45	**(1.24,1.70)*****	1.59	**(1.28,1.97)*****	0.000
CVD mortality							
	Model 1	Ref (1)	2.59	**(1.64,4.10)*****	3.38	**(1.94,5.89)*****	0.000
	Model 2	Ref (1)	2.21	**(1.41,3.49)****	2.55	**(1.50,4.34)****	0.000
	Model 3	Ref (1)	2.10	**(1.34,3.31)****	2.29	**(1.33,3.94)****	0.003
Cancer mortality							
	Model 1	Ref (1)	1.64	**(1.21,2.23)****	1.58	**(1.07,2.35)***	0.012
	Model 2	Ref (1)	1.54	**(1.12,2.11)****	1.41	(0.96,2.06)	0.052
	Model 3	Ref (1)	1.57	**(1.12,2.18)****	1.42	(0.95,2.13)	0.061

[a]Model 1 was adjusted for age, sex, and ethnicity. Model 2 further adjusted for BMI, cotinine, exercise, education, and income. Model 3 further adjusted for nutrient supplement use, AHEI, self-reported cancer, self-reported cardiovascular diseases, self-reported hypertension, and self-reported diabetes.

[b]Data were listed as the weighted hazard ratio estimates and 95% confidence intervals calculated using cox proportional hazards regression models, with *p < 0.05, **p < 0.01, ***p < 0.001.

[c]Ref, reference.

[d]Tests for trends based on the variables containing the median values for each group.

Supplementary table S10. Association of DII with mortality in competing risk models

		DII							
		Q1[c]	Q2		Q3		Q4		P_{trend}
			HR[b]	95% CI	HR	95% CI	HR	95% CI	
CVD mortality									
	Model 1[a]	Ref (1)[d]	1.64	**(1.23,2.20)***	1.83	**(1.38,2.44)***	1.97	**(1.47,2.63)***	0.000[e]
	Model 2	Ref (1)	1.51	**(1.12,2.02)**	1.53	**(1.14,2.05)**	1.58	**(1.17,2.13)**	0.003
	Model 3	Ref (1)	1.42	**(1.05,1.92)*	1.40	**(1.04,1.90)*	1.44	**(1.05,1.98)*	0.030
Cancer mortality									
	Model 1	Ref (1)	1.08	(0.82,1.42)	1.29	(0.98,1.68)	1.30	(0.99,1.71)	0.032
	Model 2	Ref (1)	1.05	(0.80,1.38)	1.18	(0.90,1.56)	1.16	(0.88,1.53)	0.210
	Model 3	Ref (1)	1.05	(0.80,1.39)	1.19	(0.90,1.57)	1.15	(0.87,1.52)	0.250

[a]Model 1 was adjusted for age, sex, and ethnicity. Model 2 further adjusted for BMI, cotinine, exercise, education, and income. Model 3 further adjusted for nutrient supplement use, AHEI, self-reported cancer, self-reported cardiovascular diseases, self-reported hypertension, and self-reported diabetes.

[b]Data were listed as the hazard ratio estimates and 95% confidence intervals calculated by Fine and Gray's subdistribution hazards regression models, with *p < 0.05, **p < 0.01, ***p < 0.001.

[c]Q, quintile.

[d]Ref, reference.

[e]Tests for trends based on the variables containing the median values for each quartile.

Supplementary table S11. Association of DOBS with mortality in competing risk models

		DOBS							
		Q1[c]	Q2		Q3		Q4		P_{trend}
			HR[b]	95% CI	HR	95% CI	HR	95% CI	
CVD mortality									
	Model 1[a]	Ref (1)[d]	0.90	(0.76,1.06)	0.88	(0.73,1.05)	0.58	**(0.47,0.71)***	0.000[e]
	Model 2	Ref (1)	0.94	(0.80,1.11)	0.96	(0.80,1.15)	0.68	**(0.55,0.83)***	0.001
	Model 3	Ref (1)	1.00	(0.83,1.19)	1.06	(0.85,1.33)	0.81	(0.61,1.07)	0.340
Cancer mortality									
	Model 1	Ref (1)	0.91	(0.75,1.09)	1.02	(0.84,1.25)	0.86	(0.70,1.06)	0.310
	Model 2	Ref (1)	0.96	(0.79,1.16)	1.09	(0.89,1.34)	0.98	(0.79,1.22)	0.850
	Model 3	Ref (1)	0.96	(0.79,1.18)	1.07	(0.85,1.36)	0.97	(0.72,1.30)	1.000

[a]Model 1 was adjusted for age, sex, and ethnicity. Model 2 further adjusted for BMI, cotinine, exercise, education, and income. Model 3 further adjusted for total intake of energy, nutrient supplement use, AHEI, self-reported cancer, self-reported cardiovascular diseases, self-reported hypertension, and self-reported diabetes.

[b]Data were listed as the hazard ratio estimates and 95% confidence intervals calculated by Fine and Gray's subdistribution hazards regression models, with *p < 0.05, **p < 0.01, ***p < 0.001.

[c]Q, quintile.

[d]Ref, reference.

[e]Tests for trends based on the variables containing the median values for each quartile.

Supplementary table S12. Association of the combination of DII and DOBS with cardiovascular disease (CVD), and cancer mortality in competing risk models

		The combination of DII and DOBS				
		Anti-inflammatory and antioxidant diet	Composite diet category		Proinflammatory and prooxidative diet	
			HR[b]	95% CI	HR	95% CI
CVD mortality						
	Model 1[a]	Ref (1)[c]	1.91	**(1.40,2.60)*****	2.42	**(1.70,3.43)*****
	Model 2	Ref (1)	1.67	**(1.22,2.29)****	1.95	**(1.36,2.79)*****
	Model 3	Ref (1)	1.61	**(1.17,2.21)****	1.83	**(1.26,2.65)****
Cancer mortality						
	Model 1	Ref (1)	1.23	（0.95,1.60）	1.22	(0.89,1.68)
	Model 2	Ref (1)	1.14	(0.88,1.49)	1.06	(0.77,1.46)
	Model 3	Ref (1)	1.14	(0.87,1.48)	1.03	(0.74,1.43)

[a]Model 1 was adjusted for age, sex, and ethnicity. Model 2 further adjusted for BMI, cotinine, exercise, education, and income. Model 3 further adjusted for nutrient supplement use, AHEI, self-reported cancer, self-reported cardiovascular diseases, self-reported hypertension, and self-reported diabetes.

[b]Data were listed as the hazard ratio estimates and its 95% confidence intervals calculated by Fine and Gray's subdistribution hazards regression models, with *p < 0.05, **p < 0.01, ***p < 0.001.

[c]Ref, reference.

Supplementary table S13. Association of the DII with all-cause mortality stratified by age, sex, race, BMI, smoking and exercise status

			DII								
			Q1[c]	Q2		Q3		Q4		P_{trend}	$P_{interaction}$
				HR[b]	95% CI	HR	95% CI	HR	95% CI		
Age											
	Model[a]	> 45	Ref (1)[d]	0.88	(0.46,1.70)	1.06	(0.57,1.98)	1.23	(0.65,2.33)	0.545[e]	0.192
		≤ 45	Ref (1)	1.42	**(1.15,1.75)****	1.95	**(1.60,2.38)*****	2.38	**(1.98,2.85)*****	0.000	
	Mode2	> 45	Ref (1)	0.76	(0.40,1.45)	0.83	(0.43,1.60)	0.77	**(0.38,1.55)**	0.514	0.196
		≤ 45	Ref (1)	1.36	**(1.11,1.67)****	1.74	**(1.42,2.14)*****	2.00	**(1.67,2.41)*****	0.000	
	Mode3	> 45	Ref (1)	0.73	(0.37,1.43)	0.79	(0.41,1.54)	0.71	(0.35,1.46)	0.394	0.253
		≤ 45	Ref (1)	1.36	**(1.10,1.67)****	1.70	**(1.36,2.13)*****	1.97	**(1.62,2.41)*****	0.000	
Sex											
	Model	Male	Ref (1)	1.29	**(1.03,1.63)***	1.85	**(1.48,2.32)*****	2.07	**(1.65,2.59)*****	0.000	-
		Female	Ref (1)	1.28	(0.97,1.69)	1.52	**(1.18,1.95)****	1.79	**(1.41,2.29)*****	0.000	
	Mode2	Male	Ref (1)	1.21	(0.97,1.53)	1.60	**(1.27,2.03)*****	1.72	**(1.36,2.17)*****	0.000	-
		Female	Ref (1)	1.22	(0.93,1.61)	1.32	**(1.01,1.72)***	1.45	**(1.11,1.89)****	0.009	
	Mode3	Male	Ref (1)	1.21	(0.95,1.53)	1.54	**(1.20,1.95)****	1.68	**(1.30,2.17)*****	0.000	-
		Female	Ref (1)	1.17	(0.89,1.53)	1.18	(0.89,1.58)	1.25	(0.94,1.66)	0.166	
Race											
	Model	Non-hispanic white	Ref (1)	1.28	**(1.03,1.61)***	1.77	**(1.43,2.20)*****	2.16	**(1.75,2.67)*****	0.000	-
		Others	Ref (1)	1.31	(0.97,1.77)	1.45	**(1.08,1.94)***	1.40	**(1.04,1.87)***	0.017	
	Mode2	Non-hispanic white	Ref (1)	1.21	(0.97,1.50)	1.51	**(1.19,1.92)****	1.76	**(1.42,2.17)*****	0.000	-
		Others	Ref (1)	1.25	(0.92,1.69)	1.32	(0.97,1.80)	1.21	(0.90,1.64)	0.182	
	Mode3	Non-hispanic white	Ref (1)	1.19	(0.96,1.48)	1.43	**(1.11,1.85)****	1.66	**(1.33,2.08)*****	0.000	-
		Others	Ref (1)	1.17	(0.86,1.59)	1.19	(0.87,1.63)	1.05	(0.77,1.42)	0.756	

BMI											
	Model	≤ 30	Ref (1)	1.26	**(1.00,1.59)***	1.74	**(1.42,2.14)*****	2.02	**(1.64,2.48)*****	0.000	0.956
		> 30	Ref (1)	1.34	(0.97,1.85)	1.61	**(1.16,2.24)****	1.81	**(1.32,2.49)*****	0.000	
	Mode2	≤ 30	Ref (1)	1.20	(0.96,1.50)	1.49	**(1.20,1.86)****	1.64	**(1.34,2.00)*****	0.000	0.669
		> 30	Ref (1)	1.25	(0.91,1.72)	1.45	**(1.03,2.04)***	1.56	**(1.14,2.14)****	0.004	
	Mode3	≤ 30	Ref (1)	1.17	(0.94,1.47)	1.39	**(1.11,1.74)****	1.47	**(1.19,1.81)*****	0.000	0.809
		> 30	Ref (1)	1.22	(0.87,1.69)	1.35	(0.94,1.94)	1.50	**(1.05,2.13)***	0.022	
Smoking											
	Model	Yes	Ref (1)	0.87	(0.58,1.31)	1.20	(0.83,1.73)	1.39	(0.99,1.96)	0.028	-
		No	Ref (1)	1.50	**(1.19,1.88)****	1.88	**(1.53,2.30)*****	1.98	**(1.59,2.46)*****	0.000	
	Mode2	Yes	Ref (1)	0.84	(0.55,1.27)	1.15	(0.79,1.68)	1.30	(0.89,1.89)	0.101	-
		No	Ref (1)	1.43	**(1.15,1.79)****	1.68	**(1.36,2.07)*****	1.71	**(1.38,2.12)*****	0.000	
	Mode3	Yes	Ref (1)	0.85	(0.56,1.29)	1.12	(0.78,1.61)	1.24	(0.85,1.79)	0.160	-
		No	Ref (1)	1.40	**(1.11,1.75)****	1.56	**(1.24,1.97)*****	1.60	**(1.27,2.02)*****	0.000	
Exercise											
	Model	Yes	Ref (1)	1.13	(0.77,1.66)	1.60	**(1.13,2.25)****	1.55	**(1.10,2.19)***	0.002	-
		No	Ref (1)	1.52	(0.65,3.53)	2.09	(0.97,4.48)	1.80	(0.65,4.93)	0.089	
	Mode2	Yes	Ref (1)	1.13	(0.76,1.67)	1.54	**(1.05,2.24)***	1.48	**(1.03,2.11)***	0.009	-
		No	Ref (1)	1.47	(0.62,3.52)	1.82	(0.85,3.90)	1.59	(0.55,4.62)	0.194	
	Mode3	Yes	Ref (1)	1.12	(0.76,1.67)	1.57	**(1.08,2.27)***	1.51	**(1.04,2.18)***	0.007	-
		No	Ref (1)	1.39	(0.55,3.54)	1.89	(0.79,4.49)	1.84	(0.54,6.31)	0.200	

[a]The variables adjusted in each model were the factors mentioned above except the stratification variables.

[b]Data were listed as the weighted hazard ratio estimates and 95% confidence intervals calculated using cox proportional hazards regression models, with *p < 0.05, **p < 0.01, ***p < 0.001.

[c]Q, quintile.

[d]Ref, reference.

[e]Tests for trends based on the variables containing the median values for each quartile.

Supplementary table S14. Association of DII with CVD mortality stratified by age, sex, race, BMI, smoking and exercise status

			DII							P_{trend}	$P_{interaction}$
			Q1[c]	Q2		Q3		Q4			
				HR[b]	95% CI	HR	95% CI	HR	95% CI		
Age											
	Model[a]	> 45	Ref (1)[d]	0.44	(0.09,2.20)	0.49	(0.11,2.24)	1.17	(0.26,5.22)	0.986[e]	0.081
		≤ 45	Ref (1)	1.82	**(1.25,2.67)****	2.79	**(1.90,4.10)*****	3.06	**(2.10,4.45)*****	0.000	
	Mode2	> 45	Ref (1)	0.35	(0.07,1.82)	0.34	(0.07,1.64)	0.64	(0.12,3.44)	0.583	0.085
		≤ 45	Ref (1)	1.71	**(1.18,2.49)****	2.35	**(1.61,3.44)*****	2.45	**(1.73,3.47)*****	0.000	
	Mode3	> 45	Ref (1)	0.31	(0.06,1.58)	0.30	(0.06,1.47)	0.53	(0.10,2.73)	0.455	0.092
		≤ 45	Ref (1)	1.68	**(1.17,2.42)****	2.19	**(1.49,3.22)*****	2.31	**(1.59,3.34)*****	0.000	
Sex											
	Model	Male	Ref (1)	1.54	(0.92,2.58)	2.24	**(1.37,3.67)****	2.50	**(1.52,4.11)*****	0.000	-
		Female	Ref (1)	1.62	(0.85,3.07)	2.25	**(1.25,4.07)****	2.22	**(1.29,3.81)****	0.000	
	Mode2	Male	Ref (1)	1.40	(0.85,2.32)	1.82	**(1.14,2.90)***	2.00	**(1.24,3.23)****	0.002	-
		Female	Ref (1)	1.43	(0.75,2.73)	1.72	(0.95,3.13)	1.56	(0.89,2.72)	0.065	
	Mode3	Male	Ref (1)	1.34	(0.81,2.23)	1.62	(0.98,2.67)	1.82	**(1.10,2.99)***	0.013	-
		Female	Ref (1)	1.32	(0.69,2.52)	1.45	(0.78,2.71)	1.33	(0.77,2.28)	0.252	
Race											
	Model	Non-hispanic white	Ref (1)	1.56	**(1.04,2.33)***	2.42	**(1.55,3.77)*****	2.64	**(1.65,4.24)*****	0.000	-
		Others	Ref (1)	1.51	(0.75,3.04)	1.53	(0.86,2.70)	1.47	(0.70,3.08)	0.261	
	Mode2	Non-hispanic white	Ref (1)	1.39	(0.94,2.07)	1.88	**(1.21,2.90)****	2.01	**(1.28,3.17)****	0.001	-
		Others	Ref (1)	1.40	(0.69,2.84)	1.33	(0.74,2.36)	1.19	(0.55,2.55)	0.699	
	Mode3	Non-hispanic white	Ref (1)	1.37	(0.94,2.02)	1.73	**(1.11,2.70)***	1.91	**(1.22,2.99)****	0.003	-

		Others	Ref (1)	1.24	(0.61,2.52)	1.11	(0.61,2.01)	0.91	(0.42,1.97)	0.655	
BMI											
	Model	≤ 30	Ref (1)	1.39	(0.86,2.27)	2.01	(1.33,3.03)**	2.23	(1.41,3.54)**	0.000	0.771
		> 30	Ref (1)	1.78	(0.87,3.65)	2.35	(1.09,5.07)*	2.28	(1.09,4.74)*	0.010	
	Mode2	≤ 30	Ref (1)	1.30	(0.81,2.10)	1.67	(1.09,2.56)*	1.78	(1.15,2.75)*	0.006	0.578
		> 30	Ref (1)	1.64	(0.79,3.40)	2.06	(0.95,4.46)	1.90	(0.90,4.04)	0.047	
	Mode3	≤ 30	Ref (1)	1.24	(0.78,1.98)	1.43	(0.92,2.22)	1.45	(0.94,2.25)	0.068	0.576
		> 30	Ref (1)	1.60	(0.77,3.31)	1.91	(0.88,4.15)	1.84	(0.85,3.95)	0.078	
Smoking											
	Model	Yes	Ref (1)	1.25	(0.48,3.25)	1.79	(0.70,4.62)	2.04	(0.87,4.79)	0.061	-
		No	Ref (1)	1.59	(1.07,2.38)*	2.34	(1.59,3.44)***	2.23	(1.50,3.31)***	0.000	
	Mode2	Yes	Ref (1)	1.07	(0.42,2.71)	1.45	(0.58,3.66)	1.64	(0.68,3.94)	0.203	-
		No	Ref (1)	1.46	(0.98,2.17)	1.90	(1.31,2.77)**	1.73	(1.18,2.54)**	0.001	
	Mode3	Yes	Ref (1)	1.09	(0.41,2.92)	1.21	(0.48,3.05)	1.33	(0.54,3.28)	0.502	-
		No	Ref (1)	1.40	(0.94,2.09)	1.73	(1.15,2.61)**	1.60	(1.07,2.39)*	0.008	
Exercise											
	Model	Yes	Ref (1)	1.40	(0.92,2.11)	1.98	(1.30,3.02)**	2.01	(1.30,3.10)**	0.000	-
		No	Ref (1)	3.03	(1.07,8.56)*	3.30	(0.76,14.26)	5.16	(1.60,16.67)**	0.004	
	Mode2	Yes	Ref (1)	1.32	(0.88,1.98)	1.72	(1.14,2.60)*	1.68	(1.10,2.56)*	0.006	-
		No	Ref (1)	2.67	(0.98,7.25)	2.04	(0.58,7.17)	2.71	(0.62,11.94)	0.177	
	Mode3	Yes	Ref (1)	1.26	(0.85,1.88)	1.52	(1.00,2.31)*	1.48	(0.97,2.27)	0.040	-
		No	Ref (1)	2.55	(0.91,7.17)	1.67	(0.46,6.02)	2.56	(0.55,11.90)	0.238	

[a]The variables adjusted in each model were the factors mentioned above except the stratification variables.

[b]Data were listed as the weighted hazard ratio estimates and 95% confidence intervals calculated using cox proportional hazards regression models, with *p < 0.05, **p < 0.01, ***p < 0.001.

[c]Q, quintile.

[d]Ref, reference.

[e]Tests for trends based on the variables containing the median values for each quartile.

Supplementary table S15. Association of the DII with cancer mortality stratified by sex, race, BMI, smoking and exercise status

			DII							P_{trend}	$P_{interaction}$
			Q1[c]	Q2		Q3		Q4			
				HR[b]	95% CI	HR	95% CI	HR	95% CI		
Sex											
	Model[a]	Male	Ref (1)[d]	1.15	(0.77,1.71)	2.15	**(1.47,3.13)*** **	1.65	**(1.04,2.62)***	0.001[e]	-
		Female	Ref (1)	1.21	(0.71,2.09)	1.15	(0.66,2.01)	1.45	(0.90,2.38)	0.161	
	Mode2	Male	Ref (1)	1.13	(0.75,1.70)	2.02	**(1.37,3.00)** **	1.53	(0.98,2.40)	0.002	-
		Female	Ref (1)	1.21	(0.72,2.06)	1.07	(0.60,1.90)	1.27	(0.76,2.13)	0.475	
	Mode3	Male	Ref (1)	1.16	(0.77,1.75)	2.16	**(1.43,3.25)*** **	1.65	**(1.06,2.58)***	0.001	-
		Female	Ref (1)	1.20	(0.72,2.00)	1.11	(0.65,1.89)	1.22	(0.73,2.03)	0.517	
Race											
	Model	Non-hispanic white	Ref (1)	1.15	(0.79,1.67)	1.70	**(1.19,2.42)** **	1.72	**(1.13,2.60)***	0.001	-
		Others	Ref (1)	1.56	(0.84,2.93)	1.82	**(1.02,3.25)***	1.58	(1.00,2.50)	0.035	
	Mode2	Non-hispanic white	Ref (1)	1.13	(0.78,1.64)	1.57	**(1.07,2.29)***	1.54	**(1.02,2.33)***	0.010	-
		Others	Ref (1)	1.56	(0.82,2.97)	1.81	(0.98,3.35)	1.56	(0.97,2.51)	0.040	
	Mode3	Non-hispanic white	Ref (1)	1.15	(0.79,1.68)	1.63	**(1.11,2.39)***	1.60	**(1.03,2.48)***	0.009	-
		Others	Ref (1)	1.54	(0.81,2.93)	1.84	(0.99,3.40)	1.48	(0.92,2.38)	0.066	
BMI											
	Model	≤ 30	Ref (1)	1.25	(0.83,1.88)	2.22	**(1.54,3.19)*** **	1.89	**(1.15,3.10)***	0.001	0.574
		> 30	Ref (1)	1.07	(0.62,1.84)	0.97	(0.57,1.65)	1.21	(0.75,1.96)	0.542	
	Mode2	≤ 30	Ref (1)	1.23	(0.82,1.84)	2.03	**(1.40,2.96)*** **	1.75	**(1.06,2.89)***	0.003	0.681
		> 30	Ref (1)	1.01	(0.58,1.75)	0.87	(0.49,1.52)	1.05	(0.65,1.68)	0.969	
	Mode3	≤ 30	Ref (1)	1.23	(0.83,1.83)	2.06	**(1.43,2.98)*** **	1.69	(0.99,2.88)	0.005	0.567
		> 30	Ref (1)	1.01	(0.59,1.71)	0.87	(0.47,1.61)	1.19	(0.72,1.95)	0.712	

Smoking											
	Model	Yes	Ref (1)	0.68	(0.34,1.36)	1.05	(0.52,2.11)	1.08	(0.58,2.02)	0.641	-
		No	Ref (1)	1.57	**(1.03,2.41)***	1.90	**(1.23,2.93)****	1.66	**(1.05,2.61)***	0.006	
	Mode2	Yes	Ref (1)	0.70	(0.35,1.41)	1.11	(0.53,2.31)	1.14	(0.60,2.16)	0.558	-
		No	Ref (1)	1.59	**(1.05,2.41)***	1.92	**(1.27,2.91)****	1.70	**(1.09,2.64)***	0.003	
	Mode3	Yes	Ref (1)	0.71	(0.35,1.46)	1.13	(0.56,2.27)	1.16	(0.60,2.23)	0.518	-
		No	Ref (1)	1.63	**(1.09,2.44)***	2.03	**(1.37,3.01)****	1.83	**(1.20,2.80)****	0.000	
Exercise											
	Model	Yes	Ref (1)	1.13	(0.77,1.66)	1.60	**(1.13,2.25)****	1.55	**(1.10,2.19)***	0.002	-
		No	Ref (1)	1.52	(0.65,3.53)	2.09	(0.97,4.48)	1.80	(0.65,4.93)	0.089	
	Mode2	Yes	Ref (1)	1.13	(0.76,1.67)	1.54	**(1.05,2.24)***	1.48	**(1.03,2.11)***	0.009	-
		No	Ref (1)	1.47	(0.62,3.52)	1.82	(0.85,3.90)	1.59	(0.55,4.62)	0.194	
	Mode3	Yes	Ref (1)	1.12	(0.76,1.67)	1.57	**(1.08,2.27)***	1.51	**(1.04,2.18)***	0.007	-
		No	Ref (1)	1.39	(0.55,3.54)	1.89	(0.79,4.49)	1.84	(0.54,6.31)	0.200	

[a]The variables adjusted in each model were the factors mentioned above except the stratification variables.

[b]Data were listed as the weighted hazard ratio estimates and 95% confidence intervals calculated using cox proportional hazards regression models, with *p < 0.05, **p < 0.01, ***p < 0.001.

[c]Q, quintile.

[d]Ref, reference.

[e]Tests for trends based on the variables containing the median values for each quartile.

Supplementary table S16. Association of the DOBS with all-cause mortality stratified by age, sex, race, BMI, smoking and exercise status

			DOBS								
			Q1[c]	Q2		Q3		Q4		P_{trend}	$P_{interaction}$
				HR[b]	95% CI	HR	95% CI	HR	95% CI		
Age											
	Model[a]	> 45	Ref (1)[d]	0.60	**(0.38,0.96)***	0.77	(0.47,1.27)	0.36	**(0.20,0.66)****	0.002[e]	0.016
		≤ 45	Ref (1)	0.79	**(0.70,0.90)*****	0.66	**(0.59,0.74)*****	0.47	**(0.41,0.53)*****	0.00	
	Mode2	> 45	Ref (1)	0.69	(0.43,1.11)	1.00	(0.60,1.67)	0.54	(0.28,1.04)	0.125	0.010
		≤ 45	Ref (1)	0.87	**(0.77,0.98)***	0.76	**(0.68,0.85)*****	0.58	**(0.51,0.65)*****	0.000	
	Mode3	> 45	Ref (1)	0.72	(0.44,1.19)	1.11	(0.60,2.04)	0.64	(0.28,1.45)	0.477	0.010
		≤ 45	Ref (1)	0.95	(0.84,1.09)	0.89	(0.78,1.02)	0.76	**(0.65,0.90)****	0.003	
Sex											
	Model	Male	Ref (1)	0.87	(0.72,1.04)	0.75	**(0.62,0.90)****	0.57	**(0.47,0.68)*****	0.000	-
		Female	Ref (1)	0.80	**(0.70,0.90)*****	0.81	**(0.67,0.97)***	0.50	**(0.42,0.60)*****	0.000	
	Mode2	Male	Ref (1)	0.93	(0.77,1.13)	0.84	(0.69,1.01)	0.68	**(0.57,0.81)*****	0.000	-
		Female	Ref (1)	0.89	**(0.79,1.00)***	0.93	(0.77,1.13)	0.62	**(0.51,0.75)*****	0.000	
	Mode3	Male	Ref (1)	0.91	(0.74,1.13)	0.81	(0.65,1.03)	0.66	**(0.52,0.85)****	0.002	-
		Female	Ref (1)	0.98	(0.85,1.12)	1.08	(0.87,1.34)	0.78	(0.60,1.01)	0.322	
Race											
	Model	Non-hispanic white	Ref (1)	0.77	**(0.67,0.88)*****	0.69	**(0.60,0.79)*****	0.50	**(0.43,0.58)*****	0.000	-
		Others	Ref (1)	0.97	(0.82,1.15)	1.10	(0.89,1.36)	0.72	**(0.60,0.88)****	0.035	
	Mode2	Non-hispanic white	Ref (1)	0.84	**(0.73,0.95)****	0.77	**(0.66,0.88)*****	0.60	**(0.52,0.69)*****	0.000	-
		Others	Ref (1)	1.03	(0.88,1.22)	1.22	(0.98,1.52)	0.83	(0.68,1.01)	0.565	
	Mode3	Non-hispanic white	Ref (1)	0.86	**(0.74,0.99)***	0.80	**(0.66,0.96)***	0.65	**(0.52,0.80)*****	0.000	-
		Others	Ref (1)	1.09	(0.90,1.32)	1.36	**(1.05,1.76)***	1.00	(0.75,1.32)	0.312	

BMI											
	Model	≤ 30	Ref (1)	0.79	**(0.69,0.90)***	0.74	**(0.64,0.86)***	0.54	**(0.47,0.63)***	0.000	0.378
		> 30	Ref (1)	0.90	(0.74,1.10)	0.84	(0.69,1.02)	0.55	**(0.44,0.69)***	0.000	
	Mode2	≤ 30	Ref (1)	0.87	**(0.76,1.00)***	0.85	**(0.73,1.00)***	0.67	**(0.58,0.78)***	0.000	0.807
		> 30	Ref (1)	0.96	(0.79,1.16)	0.91	(0.75,1.09)	0.63	**(0.51,0.79)***	0.000	
	Mode3	≤ 30	Ref (1)	0.90	(0.78,1.04)	0.88	(0.73,1.07)	0.72	**(0.58,0.89)****	0.007	0.705
		> 30	Ref (1)	0.98	(0.79,1.22)	0.96	(0.74,1.24)	0.71	**(0.50,1.00)***	0.114	
Smoking											
	Model	Yes	Ref (1)	1.11	(0.84,1.45)	0.94	(0.71,1.25)	0.75	**(0.58,0.96)***	0.043	-
		No	Ref (1)	0.79	**(0.69,0.89)***	0.77	**(0.68,0.87)***	0.55	**(0.48,0.64)***	0.000	
	Mode2	Yes	Ref (1)	1.13	(0.87,1.46)	1.00	(0.76,1.31)	0.80	(0.63,1.02)	0.130	-
		No	Ref (1)	0.83	**(0.73,0.94)****	0.84	**(0.74,0.95)****	0.63	**(0.54,0.72)***	0.000	
	Mode3	Yes	Ref (1)	1.13	(0.84,1.52)	1.05	(0.74,1.49)	0.86	(0.59,1.25)	0.660	-
		No	Ref (1)	0.87	(0.776,1.01)	0.91	(0.76,1.07)	0.73	**(0.59,0.89)****	0.008	
Exercise											
	Model	Yes	Ref (1)	0.87	**(0.76,0.99)***	0.83	**(0.71,0.97)***	0.56	**(0.48,0.65)***	0.000	-
		No	Ref (1)	0.74	**(0.57,0.97)***	0.65	**(0.48,0.86)****	0.56	**(0.45,0.69)***	0.000	
	Mode2	Yes	Ref (1)	0.93	(0.82,1.05)	0.91	(0.79,1.06)	0.65	**(0.57,0.75)***	0.000	-
		No	Ref (1)	0.80	(0.62,1.03)	0.72	**(0.53,0.97)***	0.66	**(0.53,0.83)***	0.000	
	Mode3	Yes	Ref (1)	0.95	(0.83,1.09)	0.95	(0.78,1.15)	0.70	**(0.56,0.87)****	0.012	-
		No	Ref (1)	0.80	(0.62,1.04)	0.74	(0.54,1.02)	0.72	**(0.53,0.99)***	0.043	

[a]The variables adjusted in each model were the factors mentioned above except the stratification variables.

[b]Data were listed as the weighted hazard ratio estimates and 95% confidence intervals calculated using cox proportional hazards regression models, with *p < 0.05, **p < 0.01, ***p < 0.001.

[c]Q, quintile.

[d]Ref, reference.

[e]Tests for trends based on the variables containing the median values for each quartile.

Supplementary table S17. Association of the DOBS with cardiovascular disease (CVD) mortality stratified by age, sex, race, BMI, smoking and exercise status

			DOBS								
			Q1[c]	Q2		Q3		Q4		P_{trend}	$P_{interaction}$
				HR[b]	95% CI	HR	95% CI	HR	95% CI		
Age											
	Model[a]	> 45	Ref (1)[d]	0.52	(0.16,1.63)	0.94	(0.37,2.41)	0.23	**(0.06,0.80)***	0.041[e]	0.001
		≤ 45	Ref (1)	0.87	(0.71,1.06)	0.66	**(0.54,0.81)*****	0.37	**(0.28,0.49)*****	0.000	
	Mode2	> 45	Ref (1)	0.60	(0.19,1.93)	1.25	(0.46,3.41)	0.36	(0.10,1.31)	0.305	0.001
		≤ 45	Ref (1)	0.95	(0.78,1.15)	0.78	**(0.64,0.96)***	0.47	**(0.36,0.61)*****	0.000	
	Mode3	> 45	Ref (1)	0.51	(0.18,1.51)	1.20	(0.38,3.8)	0.36	(0.06,2.31)	0.484	0.002
		≤ 45	Ref (1)	1.13	(0.90,1.42)	1.04	(0.78,1.39)	0.78	(0.53,1.16)	0.370	
Sex											
	Model	Male	Ref (1)	0.96	(0.69,1.33)	0.86	(0.66,1.10)	0.49	**(0.35,0.68)*****	0.000	-
		Female	Ref (1)	0.89	(0.69,1.14)	0.77	(0.56,1.06)	0.38	**(0.25,0.57)*****	0.000	
	Mode2	Male	Ref (1)	1.03	(0.73,1.45)	0.96	(0.72,1.26)	0.59	**(0.43,0.82)****	0.000	-
		Female	Ref (1)	0.99	(0.77,1.27)	0.91	(0.66,1.26)	0.49	**(0.32,0.75)****	0.004	
	Mode3	Male	Ref (1)	1.03	(0.72,1.48)	0.99	(0.71,1.38)	0.66	(0.42,1.05)	0.083	-
		Female	Ref (1)	1.22	(0.89,1.67)	1.27	(0.81,2.00)	0.80	(0.45,1.43)	0.922	
Race											
	Model	Non-hispanic white	Ref (1)	0.91	(0.73,1.13)	0.74	**(0.59,0.92)****	0.44	**(0.33,0.58)*****	0.000	-
		Others	Ref (1)	0.83	(0.58,1.20)	1.09	(0.68,1.75)	0.43	**(0.25,0.73)****	0.038	
	Mode2	Non-hispanic white	Ref (1)	0.96	(0.77,1.20)	0.82	(0.65,1.04)	0.53	**(0.41,0.68)*****	0.000	-
		Others	Ref (1)	0.90	(0.62,1.30)	1.25	(0.76,2.07)	0.51	**(0.30,0.89)***	0.239	
	Mode3	Non-hispanic white	Ref (1)	1.02	(0.79,1.30)	0.92	(0.68,1.25)	0.65	**(0.43,0.97)***	0.055	-

		Others	Ref (1)	1.06	(0.69,1.61)	1.71	**(1.01,2.91)***	0.86	(0.40,1.82)	0.506	
BMI											
	Model	≤ 30	Ref (1)	0.94	(0.71,1.24)	0.80	(0.62,1.04)	0.49	**(0.36,0.67)*****	0.000	0.853
		> 30	Ref (1)	0.88	(0.62,1.26)	0.85	(0.60,1.21)	0.41	**(0.27,0.62)*****	0.000	
	Mode2	≤ 30	Ref (1)	1.04	(0.78,1.38)	0.92	(0.71,1.23)	0.60	**(0.45,0.82)****	0.001	0.591
		> 30	Ref (1)	0.94	(0.66,1.32)	0.92	(0.66,1.30)	0.47	**(0.31,0.71)*****	0.001	
	Mode3	≤ 30	Ref (1)	1.11	(0.81,1.54)	1.05	(0.72,1.52)	0.73	(0.45,1.19)	0.297	0.717
		> 30	Ref (1)	1.05	(0.73,1.52)	1.14	(0.73,1.78)	0.68	(0.38,1.23)	0.482	
Smoking											
	Model	Yes	Ref (1)	1.27	(0.71,2.27)	0.80	(0.48,1.33)	0.32	**(0.18,0.58)*****	0.001	-
		No	Ref (1)	0.87	(0.70,1.08)	0.85	(0.68,1.06)	0.50	**(0.39,0.65)*****	0.000	
	Mode2	Yes	Ref (1)	1.26	(0.70,2.27)	0.86	(0.51,1.46)	0.35	**(0.19,0.64)****	0.006	-
		No	Ref (1)	0.93	(0.75,1.16)	0.95	(0.75,1.19)	0.60	**(0.48,0.77)*****	0.000	
	Mode3	Yes	Ref (1)	1.11	(0.59,2.07)	0.75	(0.38,1.48)	0.29	**(0.12,0.71)****	0.053	-
		No	Ref (1)	1.08	(0.85,1.36)	1.20	(0.88,1.64)	0.90	(0.63,1.29)	0.921	
Exercise											
	Model	Yes	Ref (1)	1.00	(0.82,1.23)	0.89	(0.74,1.08)	0.48	**(0.35,0.65)*****	0.000	-
		No	Ref (1)	0.68	(0.43,1.07)	0.61	(0.37,1.02)	0.40	**(0.25,0.64)*****	0.000	
	Mode2	Yes	Ref (1)	1.05	(0.86,1.28)	0.97	(0.80,1.19)	0.55	**(0.41,0.74)*****	0.000	-
		No	Ref (1)	0.78	(0.48,1.26)	0.74	(0.42,1.29)	0.54	**(0.33,0.88)***	0.018	
	Mode3	Yes	Ref (1)	1.17	(0.93,1.47)	1.19	(0.89,1.57)	0.76	(0.49,1.18)	0.537	-
		No	Ref (1)	0.70	(0.42,1.17)	0.65	(0.31,1.35)	0.49	(0.22,1.08)	0.097	

[a]The variables adjusted in each model were the factors mentioned above except the stratification variables.

[b]Data were listed as the weighted hazard ratio estimates and 95% confidence intervals calculated using cox proportional hazards regression models, with *p < 0.05, **p < 0.01, ***p < 0.001.

[c]Q, quintile.

[d]Ref, reference.

[e]Tests for trends based on the variables containing the median values for each quartile.

Supplementary table S18. Association of the DOBS with cancer mortality stratified by sex, race, BMI, smoking and exercise status

			DOBS							P_{trend}	$P_{interaction}$
			Q1[c]	Q2		Q3		Q4			
				HR[b]	95% CI	HR	95% CI	HR	95% CI		
Sex											
	Model[a]	Male	Ref (1)[d]	0.83	(0.61,1.15)	0.85	(0.59,1.24)	0.66	**(0.46,0.95)***	0.033[e]	-
		Female	Ref (1)	0.76	(0.56,1.04)	0.91	(0.60,1.38)	0.56	**(0.37,0.84)****	0.039	
	Mode2	Male	Ref (1)	0.90	(0.65,1.24)	0.94	(0.65,1.37)	0.79	(0.55,1.14)	0.253	-
		Female	Ref (1)	0.85	(0.62,1.16)	1.04	(0.67,1.62)	0.68	(0.44,1.05)	0.291	
	Mode3	Male	Ref (1)	0.81	(0.59,1.13)	0.78	(0.51,1.20)	0.60	**(0.36,0.98)***	0.056	-
		Female	Ref (1)	0.90	(0.63,1.29)	1.15	(0.67,1.96)	0.79	(0.43,1.44)	0.753	
Race											
	Model	Non-hispanic white	Ref (1)	0.73	**(0.57,0.95)***	0.79	(0.58,1.07)	0.57	**(0.42,0.78)*****	0.002	-
		Others	Ref (1)	1.00	(0.71,1.41)	1.15	(0.74,1.81)	0.76	(0.51,1.13)	0.448	
	Mode2	Non-hispanic white	Ref (1)	0.80	(0.62,1.03)	0.87	(0.65,1.17)	0.70	**(0.52,0.95)***	0.050	-
		Others	Ref (1)	1.05	(0.74,1.49)	1.21	(0.77,1.92)	0.81	(0.53,1.23)	0.682	
	Mode3	Non-hispanic white	Ref (1)	0.78	(0.59,1.03)	0.83	(0.57,1.21)	0.66	(0.43,1.03)	0.110	-
		Others	Ref (1)	1.08	(0.73,1.59)	1.21	(0.70,2.11)	0.80	(0.41,1.57)	0.851	
BMI											
	Model	≤ 30	Ref (1)	0.77	(0.58,1.01)	0.94	(0.67,1.32)	0.61	**(0.45,0.84)****	0.016	0.573
		> 30	Ref (1)	0.85	(0.58,1.24)	0.72	(0.47,1.11)	0.65	(0.42,1.01)	0.043	
	Mode2	≤ 30	Ref (1)	0.84	(0.63,1.12)	1.04	(0.73,1.49)	0.73	(0.53,1.02)	0.195	0.335
		> 30	Ref (1)	0.90	(0.62,1.30)	0.79	(0.51,1.21)	0.76	(0.48,1.20)	0.196	
	Mode3	≤ 30	Ref (1)	0.87	(0.65,1.16)	1.04	(0.70,1.55)	0.76	(0.49,1.16)	0.385	0.397
		> 30	Ref (1)	0.80	(0.52,1.23)	0.68	(0.39,1.17)	0.61	(0.30,1.24)	0.147	

Smoking											
	Model	Yes	Ref (1)	1.44	(0.86,2.40)	1.47	(0.86,2.51)	1.23	(0.77,1.98)	0.269	-
		No	Ref (1)	0.67	**(0.50,0.89)****	0.74	(0.53,1.03)	0.59	**(0.43,0.80)****	0.003	
	Mode2	Yes	Ref (1)	1.45	(0.88,2.40)	1.48	(0.87,2.50)	1.24	(0.79,1.95)	0.235	-
		No	Ref (1)	0.68	**(0.51,0.91)****	0.77	(0.55,1.06)	0.62	**(0.45,0.85)****	0.010	
	Mode3	Yes	Ref (1)	1.54	(0.90,2.64)	1.63	(0.87,3.04)	1.49	(0.81,2.76)	0.157	-
		No	Ref (1)	0.65	**(0.48,0.87)****	0.69	(0.48,1.02)	0.53	**(0.33,0.85)****	0.013	
Exercise											
	Model	Yes	Ref (1)	0.77	**(0.60,0.99)***	0.93	(0.66,1.30)	0.66	**(0.49,0.88)****	0.032	-
		No	Ref (1)	0.90	(0.58,1.38)	0.70	(0.40,1.23)	0.57	**(0.37,0.89)***	0.010	
	Mode2	Yes	Ref (1)	0.83	(0.65,1.06)	1.02	(0.72,1.43)	0.77	(0.57,1.04)	0.243	-
		No	Ref (1)	0.93	(0.59,1.47)	0.75	(0.42,1.33)	0.64	(0.40,1.02)	0.041	
	Mode3	Yes	Ref (1)	0.79	(0.60,1.03)	0.91	(0.61,1.38)	0.66	(0.43,1.02)	0.141	-
		No	Ref (1)	1.01	(0.65,1.59)	0.88	(0.47,1.64)	0.81	(0.44,1.51)	0.477	

[a]The variables adjusted in each model were the factors mentioned above except the stratification variables.

[b]Data were listed as the weighted hazard ratio estimates and 95% confidence intervals calculated using cox proportional hazards regression models, with *p < 0.05, **p < 0.01, ***p < 0.001.

[c]Q, quintile.

[d]Ref, reference.

[e]Tests for trends based on the variables containing the median values for each quartile.

Supplementary table S19. Association of the combination of DII and DOBS with all-cause mortality stratified by age, sex, race, BMI, smoking and exercise status

			The combination of DII and DOBS					
			Anti-inflammatory and antioxidant diet	Composite diet category		Proinflammatory and prooxidative diet		P_{trend}
				HR[b]	95% CI	HR	95% CI	
Age								
	Model[a]	> 45	Ref (1)[c]	2.22	(0.99,4.98)	2.58	**(1.14,5.82)***	0.007[d]
		≤ 45	Ref (1)	1.69	**(1.43,1.99)*****	2.57	**(2.07,3.19)*****	0.000
	Mode2	> 45	Ref (1)	1.79	(0.75,4.24)	1.62	(0.66,3.99)	0.292
		≤ 45	Ref (1)	1.55	**(1.31,1.83)*****	2.10	**(1.71,2.59)*****	0.000
	Mode3	> 45	Ref (1)	1.74	(0.70,4.36)	1.55	(0.60,4.03)	0.397
		≤ 45	Ref (1)	1.59	**(1.34,1.88)*****	2.04	**(1.64,2.54)*****	0.000
Sex								
	Model	Male	Ref (1)	1.66	**(1.38,3.01)*****	2.17	**(1.63,2.89)*****	0.000
		Female	Ref (1)	1.70	**(1.35,2.16)*****	2.17	**(1.63,2.87)*****	0.000
	Mode2	Male	Ref (1)	1.49	**(1.24,1.80)*****	1.74	**(1.32,2.30)*****	0.000
		Female	Ref (1)	1.52	**(1.19,1.94)****	1.71	**(1.26,2.32)****	0.004
	Mode3	Male	Ref (1)	1.47	**(1.21,1.78)*****	1.69	**(1.25,2.29)****	0.000
		Female	Ref (1)	1.46	**(1.15,1.85)****	1.48	**(1.10,2.00)***	0.083
Race								
	Model	Non-hispanic white	Ref (1)	1.72	**(1.45,2.03)*****	2.39	**(1.84,3.10)*****	0.000
		Others	Ref (1)	1.46	**(1.08,1.97)***	1.54	**(1.12,2.11)****	0.014
	Mode2	Non-hispanic white	Ref (1)	1.53	**(1.29,1.81)*****	1.88	**(1.47,2.41)*****	0.000
		Others	Ref (1)	1.36	(1.00,1.84)	1.34	(0.97,1.84)	0.140

	Mode3	Non-hispanic white	Ref (1)	1.51	**(1.27,1.79)*** **	1.77	**(1.37,2.27)*** **	0.000
		Others	Ref (1)	1.24	(0.92,1.68)	1.15	(0.83,1.58)	0.669
BMI								
	Model	≤ 30	Ref (1)	1.62	**(1.33,1.98)*** **	2.11	**(1.63,2.72)*** **	0.000
		> 30	Ref (1)	1.76	**(1.32,2.34)*** **	2.21	**(1.50,3.27)*** **	0.000
	Mode2	≤ 30	Ref (1)	1.46	**(1.19,1.78)*** **	1.67	**(1.31,2.12)*** **	0.000
		> 30	Ref (1)	1.59	**(1.20,2.11)** **	1.87	**(1.29,2.69)** **	0.002
	Mode3	≤ 30	Ref (1)	1.41	**(1.15,1.73)** **	1.49	**(1.17,1.92)** **	0.002
		> 30	Ref (1)	1.53	**(1.13,2.08)** **	1.75	**(1.16,2.63)** **	0.012
Smoking								
	Model	Yes	Ref (1)	1.24	(0.89,1.73)	1.31	(0.91,1.88)	0.155
		No	Ref (1)	1.79	**(1.50,2.12)*** **	2.28	**(1.79,2.90)*** **	0.000
	Mode2	Yes	Ref (1)	1.19	(0.85,1.67)	1.20	(0.83,1.73)	0.357
		No	Ref (1)	1.66	**(1.39,1.97)*** **	1.95	**(1.53,2.48)*** **	0.000
	Mode3	Yes	Ref (1)	1.21	(0.86,1.69)	1.16	(0.79,1.72)	0.517
		No	Ref (1)	1.60	**(1.35,1.91)*** **	1.81	**(1.42,2.30)*** **	0.000
Exercise								
	Model	Yes	Ref (1)	1.64	**(1.37,1.98)*** **	2.08	**(1.63,2.64)*** **	0.000
		No	Ref (1)	1.53	(0.98,2.40)	1.88	**(1.09,3.24)* **	0.014
	Mode2	Yes	Ref (1)	1.51	**(1.26,1.81)*** **	1.74	**(1.39,2.18)*** **	0.000
		No	Ref (1)	1.44	(0.92,2.26)	1.63	(0.90,2.93)	0.070
	Mode3	Yes	Ref (1)	1.46	**(1.21,1.75)*** **	1.58	**(1.24,2.03)*** **	0.001
		No	Ref (1)	1.44	(0.91,2.29)	1.66	(0.94,2.92)	0.056

[a]The variables adjusted in each model were the factors mentioned above except the stratification variables.

[b]Data were listed as the weighted hazard ratio estimates and 95% confidence intervals calculated using cox proportional hazards regression

models, with *$p < 0.05$, **$p < 0.01$, ***$p < 0.001$.

[c]Ref, reference.

[d]Tests for trends based on the variables containing the median values for each quartile.

Supplementary table S20. Association of the combination of DII and DOBS with cardiovascular disease (CVD) mortality stratified by age, sex, race, BMI, smoking and exercise status

			The combination of DII and DOBS					
			Anti-inflammatory and antioxidant diet	Composite diet category		Proinflammatory and prooxidative diet		P_{trend}
				HR[b]	95% CI	HR	95% CI	
Age								
	Model[a]	> 45	Ref (1)[c]	8.57	(0.96,76.85)	17.90	**(2.08,154.19)****	0.006[d]
		≤ 45	Ref (1)	2.64	**(1.67,4.16)*****	4.11	**(2.42,7.00)*****	0.000
	Mode2	> 45	Ref (1)	6.48	(0.72,58.00)	10.56	**(1.17,95.57)***	0.062
		≤ 45	Ref (1)	2.39	**(1.52,3.73)*****	3.26	**(1.97,5.40)*****	0.000
	Mode3	> 45	Ref (1)	5.86	(0.61,56.43)	9.33	(0.98,88.69)	0.077
		≤ 45	Ref (1)	2.39	**(1.53,3.76)*****	3.06	**(1.79,5.22)*****	0.000
Sex								
	Model	Male	Ref (1)	2.24	**(1.30,3.88)****	3.26	**(1.68,6.32)****	0.000
		Female	Ref (1)	4.41	**(1.74,11.18)****	5.16	**(2.00,13.34)****	0.000
	Mode2	Male	Ref (1)	1.97	**(1.16,3.37)***	2.58	**(1.36,4.91)****	0.002
		Female	Ref (1)	3.58	**(1.40,9.17)****	3.59	**(1.37,9.40)***	0.016
	Mode3	Male	Ref (1)	1.84	**(1.07,3.17)***	2.37	**(1.23,4.58)***	0.006
		Female	Ref (1)	3.33	**(1.30,8.54)***	3.10	**(1.18,8.14)***	0.090
Race								
	Model	Non-hispanic white	Ref (1)	2.69	**(1.60,4.55)*****	3.71	**(1.90,7.25)*****	0.000
		Others	Ref (1)	2.12	**(1.27,3.53)****	2.37	**(1.19,4.73)***	0.045
	Mode2	Non-hispanic white	Ref (1)	2.29	**(1.36,3.84)****	2.80	**(1.46,5.34)****	0.001
		Others	Ref (1)	1.91	**(1.14,3.22)***	1.95	(0.95,4.01)	0.183

	Mode3	Non-hispanic white	Ref (1)	2.25	**(1.35,3.75)****	2.64	**(1.38,5.05)****	0.003
		Others	Ref (1)	1.68	**(1.01,2.79)***	1.54	(0.75,3.15)	0.526
BMI								
	Model	≤ 30	Ref (1)	2.18	**(1.20,3.95)***	2.70	**(1.38,5.30)****	0.001
		> 30	Ref (1)	3.34	**(1.57,7.11)****	4.44	**(1.82,10.85)****	0.001
	Mode2	≤ 30	Ref (1)	1.94	**(1.07,3.54)***	2.12	**(1.09,4.11)***	0.014
		> 30	Ref (1)	2.99	**(1.40,6.42)****	3.66	**(1.50,8.92)****	0.004
	Mode3	≤ 30	Ref (1)	1.85	**(1.01,3.36)***	1.79	(0.91,3.51)	0.078
		> 30	Ref (1)	2.92	**(1.35,6.34)****	3.48	**(1.41,8.61)****	0.008
Smoking								
	Model	Yes	Ref (1)	3.25	**(1.25,8.43)***	4.21	**(1.46,12.13)****	0.007
		No	Ref (1)	2.45	**(1.51,3.98)*****	2.95	**(1.64,5.30)*****	0.000
	Mode2	Yes	Ref (1)	2.78	**(1.04,7.43)***	3.38	**(1.10,10.42)***	0.053
		No	Ref (1)	2.14	**(1.31,3.50)****	2.29	**(1.28,4.09)****	0.003
	Mode3	Yes	Ref (1)	2.69	**(1.07,6.77)***	2.86	(0.92,8.92)	0.147
		No	Ref (1)	2.05	**(1.25,3.36)****	2.11	**(1.17,3.80)***	0.011
Exercise								
	Model	Yes	Ref (1)	2.34	**(1.42,3.84)****	2.84	**(1.55,5.21)****	0.000
		No	Ref (1)	3.88	**(1.20,12.60)***	9.14	**(2.47,33.81)****	0.000
	Mode2	Yes	Ref (1)	2.12	**(1.30,3.46)****	2.37	**(1.32,4.25)****	0.003
		No	Ref (1)	3.20	**(1.02,10.07)***	5.16	**(1.11,23.88)***	0.040
	Mode3	Yes	Ref (1)	2.01	**(1.24,3.26)****	2.11	**(1.16,3.82)***	0.019
		No	Ref (1)	3.12	(0.95,10.27)	5.10	**(1.12,23.15)***	0.039

[a]The variables adjusted in each model were the factors mentioned above except the stratification variables.

[b]Data were listed as the weighted hazard ratio estimates and 95% confidence intervals calculated using cox proportional hazards regression

models, with *$p < 0.05$, **$p < 0.01$, ***$p < 0.001$.

[c]Ref, reference.

[d]Tests for trends based on the variables containing the median values for each quartile.

Supplementary table S21. Association of the combination of DII and DOBS with cancer mortality stratified by sex, race, BMI, smoking and exercise status

			The combination of DII and DOBS					P_{trend}
			Anti-inflammatory and antioxidant diet	Composite diet category		Proinflammatory and prooxidative diet		
				HR[b]	95% CI	HR	95% CI	
Sex								
	Model[a]	Male	Ref (1)[c]	1.65	**(1.17,2.32)****	1.45	(0.88,2.39)	0.017[d]
		Female	Ref (1)	1.62	(0.97,2.70)	1.68	(0.85,3.33)	0.240
	Mode2	Male	Ref (1)	1.55	**(1.09,2.19)***	1.26	(0.78,2.05)	0.079
		Female	Ref (1)	1.49	(0.90,2.46)	1.41	(0.71,2.79)	0.535
	Mode3	Male	Ref (1)	1.59	**(1.11,2.28)***	1.32	(0.80,2.17)	0.060
		Female	Ref (1)	1.56	(0.95,2.57)	1.39	(0.70,2.75)	0.624
Race								
	Model	Non-hispanic white	Ref (1)	1.66	**(1.19,2.33)****	1.65	**(1.03,2.65)***	0.014
		Others	Ref (1)	1.64	(0.93,2.89)	1.54	(0.87,2.73)	0.226
	Mode2	Non-hispanic white	Ref (1)	1.53	**(1.09,2.17)***	1.40	(0.90,2.18)	0.070
		Others	Ref (1)	1.59	(0.88,2.87)	1.48	(0.82,2.67)	0.293
	Mode3	Non-hispanic white	Ref (1)	1.58	**(1.10,2.27)***	1.44	(0.90,2.28)	0.065
		Others	Ref (1)	1.54	(0.86,2.78)	1.35	(0.74,2.43)	0.535
BMI								
	Model	≤ 30	Ref (1)	1.73	**(1.21,2.49)****	1.62	(0.95,2.77)	0.036
		> 30	Ref (1)	1.46	(0.81,2.62)	1.51	(0.76,3.00)	0.227
	Mode2	≤ 30	Ref (1)	1.60	**(1.10,2.32)***	1.43	(0.83,2.44)	0.128
		> 30	Ref (1)	1.33	(0.73,2.43)	1.27	(0.64,2.54)	0.502

	Mode3	≤ 30	Ref (1)	1.59	**(1.09,2.32)***	1.34	(0.76,2.34)	0.221
		> 30	Ref (1)	1.36	(0.71,2.60)	1.42	(0.65,3.10)	0.375
Smoking								
	Model	Yes	Ref (1)	1.07	(0.66,1.73)	0.75	(0.36,1.54)	0.347
		No	Ref (1)	1.69	**(1.14,2.51)***	1.81	**(1.12,2.92)***	0.009
	Mode2	Yes	Ref (1)	1.09	(0.67,1.78)	0.76	(0.39,1.49)	0.343
		No	Ref (1)	1.70	**(1.15,2.50)****	1.82	**(1.15,2.89)***	0.006
	Mode3	Yes	Ref (1)	1.14	(0.68,1.89)	0.81	(0.39,1.68)	0.500
		No	Ref (1)	1.76	**(1.20,2.60)****	1.92	**(1.21,3.05)****	0.003
Exercise								
	Model	Yes	Ref (1)	1.53	**(1.13,2.07)****	1.49	(0.99,2.24)	0.049
		No	Ref (1)	2.13	(0.87,5.24)	1.85	(0.76,4.54)	0.060
	Mode2	Yes	Ref (1)	1.45	**(1.05,1.99)***	1.35	(0.91,2.00)	0.130
		No	Ref (1)	1.92	(0.79,4.84)	1.65	(0.60,4.56)	0.169
	Mode3	Yes	Ref (1)	1.47	**(1.06,2.04)***	1.37	(0.91,2.05)	0.130
		No	Ref (1)	1.90	(0.69,5.26)	1.73	(0.55,5.42)	0.213

[a]The variables adjusted in each model were the factors mentioned above except the stratification variables.

[b]Data were listed as the weighted hazard ratio estimates and 95% confidence intervals calculated using cox proportional hazards regression models, with *p < 0.05, **p < 0.01, ***p < 0.001.

[c]Ref, reference.

[d]Tests for trends based on the variables containing the median values for each quartile.

Supplementary figure S1. Mediation effects of CRP and RDW on the associations of DII/DOBS/the combination of DII and DOBS with all-cause, CVD, and cancer mortality. Data were presented as standardized regression coefficients after adjusting for the corresponding covariables in the cox proportional hazards regression models. β_1: indirect relation, β2: indirect relation, β_{Tot}: total relation, and β_{dir}: direct relation. *P < 0.05, **P < 0.01, ***P < 0.001.

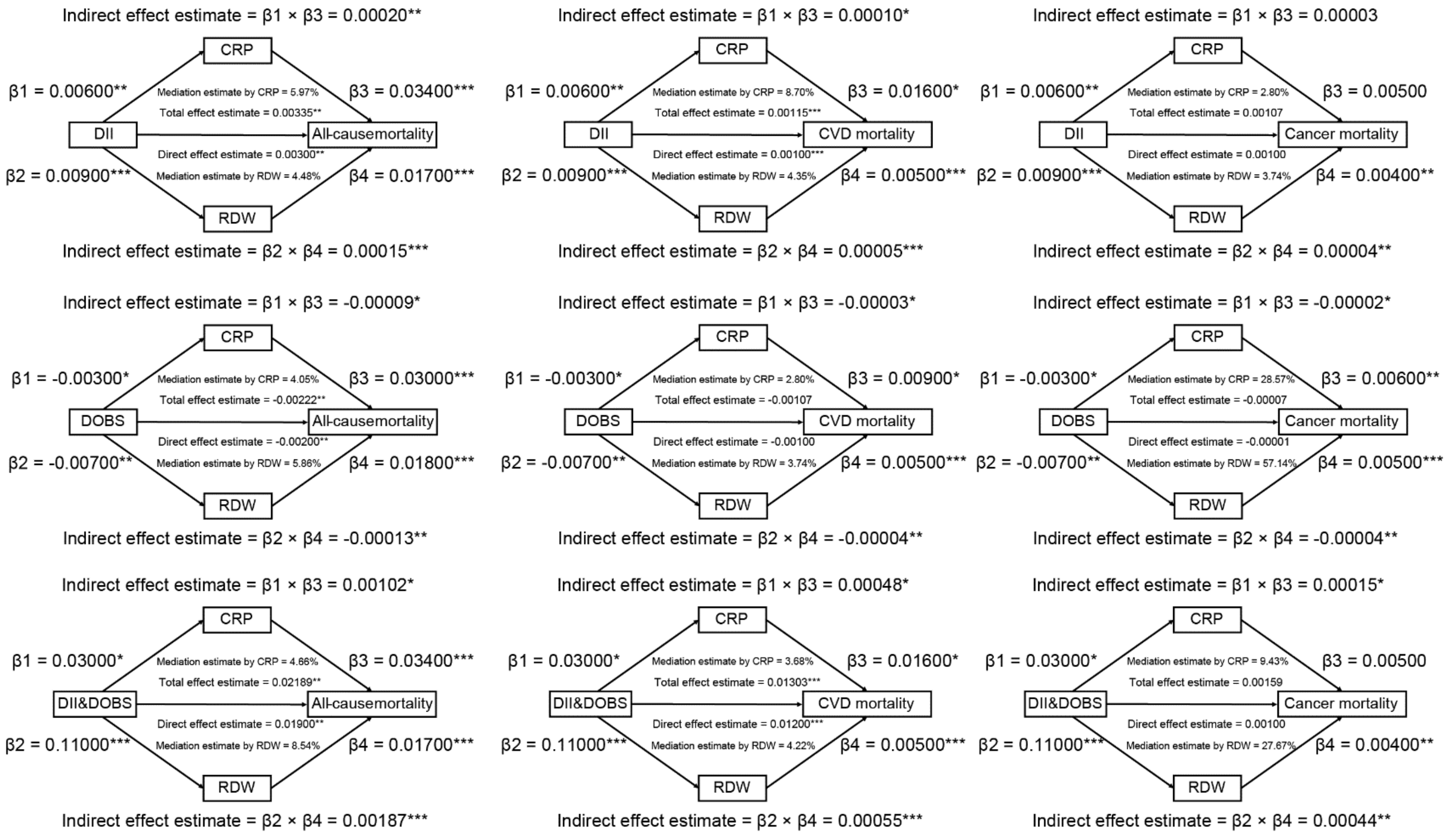

Supplement: Supplementary file 1 [file nutrients-15-03148-s001.zip › nutrients-2470141-supplementary.pdf]
